# Dense and influential core promotion of daily viral information spread in political echo chambers

**DOI:** 10.1038/s41598-021-86750-w

**Published:** 2021-04-05

**Authors:** Kimitaka Asatani, Hiroko Yamano, Takeshi Sakaki, Ichiro Sakata

**Affiliations:** 1 Graduate school of Engineering, The University of Tokyo, 7-3-1 Hongo, Bunkyo-ku, Tokyo, 113-0033 Japan; 2 Institute for Future Initiatives, The University of Tokyo, 7-3-1 Hongo, Bunkyo-ku, Tokyo, 113-0033 Japan

**Keywords:** Information technology, Computer science

## Abstract

Despite the intensive study of the viral spread of fake news in political echo chambers (ECs) on social networking services (SNSs), little is known regarding the underlying structure of the daily information spread in these ECs. Moreover, the effect of SNSs on opinion polarisation is still unclear in terms of pluralistic information access or selective exposure to opinions in an SNS. In this study, we confirmed the steady, highly independent nature of left- and right-leaning ECs, both of which are composed of approximately 250,000 users, from a year-long reply/retweet network of 42 million Japanese Twitter users. We found that both communities have similarly efficient information spreading networks with densely connected and core-periphery structures. Core nodes resonate in the early stages of information cascades, and unilaterally transmit information to peripheral nodes. Each EC has resonant core users who amplify and steadily spread information to a quarter of a million users. In addition, we confirmed the existence of extremely aggressive users of ECs who co-reply/retweet each other. The connection between these users and top influencers suggests that the extreme opinions of the former group affect the entire community through the top influencers.

## Introduction

People in political echo chambers (ECs) on social networking services (SNSs)^1,2^ leverage their beliefs, lead to extremist activities^3^, prevent political discussion among ordinary users^4^ and spread false information with hijacked hashtags^5^. Recently, the spread of fake news^6^ and conspiracy theories^7^ have become a critical issue in social well-being. Fake news and conspiracy theories diffuse rapidly and widely^8^ in SNSs, and a vast number of psychology studies and data analyses have demonstrated the virality of fake news(mis/dis/mal-information, etc) in human communication^9^. However, fake news is not the only information being spread in ECs and is not limited to ECs^10^. Therefore, the efficient information-spreading structure of ECs is also considered to enhance the propagation of information. A study^11^ has addressed the existence and potential influence of densely connected core users on Twitter in the United States. These core users may be a single-opinion influencer group because members of densely connected groups are likely to have the same opinions^12^ and attitudes^13^. However, previous analyses^11,14^ of information-spreading in ECs have been limited to specific fake news or rumours, and the structure of daily information spreading networks on ECs and the role of users are not well understood.

Moreover, despite the increased academic and industry attention on ECs, arguments still continue^15,16^ regarding the influence of social media on opinion polarisation. These studies emphasise that users in an SNS can obtain pluralistic information in a few steps^17^ – at least in principle. However, several studies have demonstrated^18,19^ opinion polarisation among ECs. Complicated discussions arise owing to the difference in the scope of user groups in these studies. Most of the studies cited herein have investigated a group of polarised people, whereas the scope of other studies included general users who retweeted or replied to certain topics^15^ or entire groups of users in a county^16^. To clarify the stationary independence and conflicting ECs, it is important to extract the ECs from user relationships not limited to a specific topic. In this study, we extracted communities from year-long reply/retweet relationships and analysed politically polarised communities.

Political polarisation has been observed in human communications on social networks in the absence of SNSs^20^. In addition, the dissemination of the attributes of a person^21^, opinion formation theories, such as the bounded confidence model^22^, and selective exposure to information^23^ can explain the emergence of homophily^24^. An agent’s cognitive and psychological behaviours that increase social conformity are factors of the evolution process that lead to group polarisation. Conformity is determined based on social balance^25^, majority in her/his neighbourhoods^26,27^, and so on. The network structures of the opinion formation, such as scale-free and small-world structures, are known to lead to faster or stronger opinion uniformity or polarisation^28–30^. However, these models do not simulate the dynamic changes in the network of opinion formation. In the segregation model^31,32^, which is another possible explanation of the polarisation, the agent dynamically changes his/her neighbours on the lattice network and intensifies the group polarisation. A recent study^33^ clarified that users adjust their opinions and connections in the simulation of co-evolution of opinions and networks. Thus, these previous studies demonstrate that the structure of a social network significantly influences opinion formation.

In the past decade, SNSs have become the central means of political discussion and the spread of information . Users on SNSs find it easy to discover the information source, resulting in a massive and scale-free spread of information^34^. Thus, the rapid spread of information^35,36^ and the existence of large ECs on SNSs are demonstrated in empirical studies^18,37,38^. Factors, such as media influence^39^, implementation of algorithms on SNSs^40,41^, and unfollowing^42^, reinforce such group biases. The simulation of opinion formation, which considers the structure of information spreading networks on SNSs, such as scale-free^28,43,44^, reveals its considerable impact on opinion formation. However, these models do not consider the details of the information spreading network, which is only partly discussed in empirical studies^8,9^. In this study, we assume that densely connected highly influential cores exist in ECs. The existence of influential nodes is known to lead to efficient information diffusion^45^. The core-connected network structure is the primary feature of the onion-like structure^46^, which is resilient to attacks and promotes efficient information diffusion^47^. Recent studies have shown that bias reinforcement in SNSs can be explained by repeated cascades of information^48^. Therefore, to clarify the daily information structure of ECs it is important to understand the existence and virality of fake news in ECs.

We found that opposing political ECs on Twitter in Japan have core-periphery structures that efficiently and unilaterally diffuse information from small-core users(influencers), based on the analysis of the network structures of left- and right-leaning ECs composed of 251,000 and 270,000 users, respectively. The influencers play a vital role in the spread of negative sentiment information. These ECs are detected from the reply/retweet relationships of 42 million users based on 10% of the Japanese tweets over 396 days. The results indicate that the EC is not overstated^16^ for the half-million users on Japanese Twitter; most of the information conveys a negative sentiment and steadily originates from the EC. Similarly, both ECs have denser and more core-periphery information spreading structures than those of most other communities, such as online games and regional communities. In both ECs, the connected core users unilaterally broadcast their news interpretations and criticisms of opposing communities to the periphery. The major information cascades, which are likely to have a greater negative sentiment than that of the other information in the ECs, resonate in the core nodes in the early information cascade stage. Our results confirm the importance of core nodes for information spreading in the EC; core nodes are partly discussed in previous studies^11,14^. Moreover, a detailed investigation of the network of core nodes suggests that the group of top influencers is tightly linked to the aggressive and extremely polarised co-reply/retweet core members who reply/retweet each other. This result suggests that the existence of extremely aggressive opinions in ECs does not significantly spread but affects the communities via the top influencers. Thus, monitoring such active groups of influencers and co-connected cores is useful in observing and controlling steadily and enthusiastic ECs.

## Results

### Independent and steady political echo chambers

**Figure 1:**
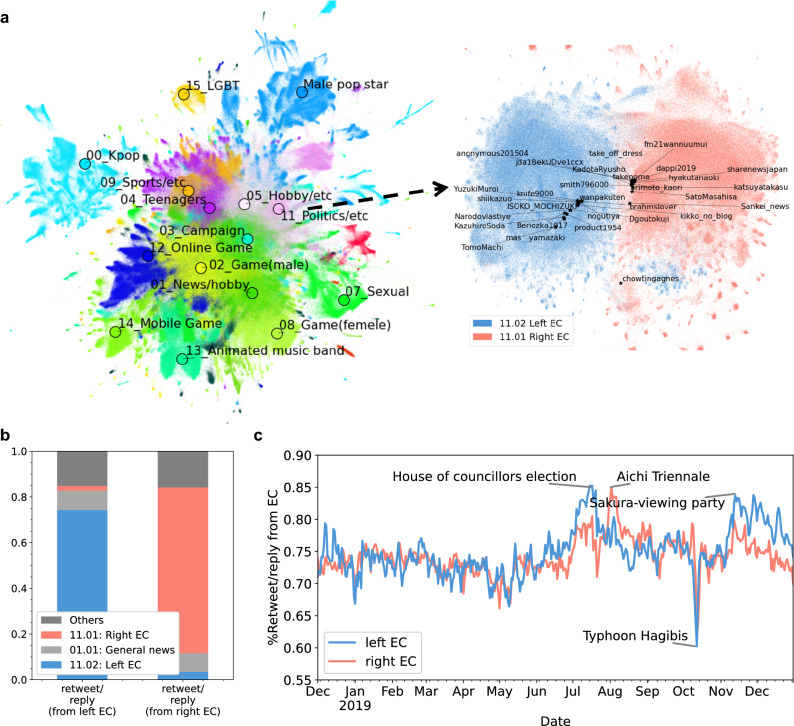
(**a**) left Visualisation of 42 million Japanese Twitter users. Each dot corresponds to a user coloured according to his/her cluster. Users are embedded in a 2D space with LINE^50^ (network representation learning) and UMAP^51^ (dimension reduction). The manually annotated labels of the top 15 clusters based on the number of users are plotted. (**a**), right Visualisation of the left- and right-leaning political ECs: The users in subcluster 11.02 (left EC) are coloured blue, and those in 11.01 (right EC) are coloured red. The top 20 influencers of each subcluster are plotted with their user names. (**b**) Ratio of clusters containing the origins of retweets/replies from the left- and right-leaning ECs. **c** Daily internal information ratio (the ratio of the origins of retweets/replies made by users of the EC in the same cluster).

Approximately 42 million Japanese Twitter users were divided into clusters using the Leiden network clustering method^49^, based on their reply/retweet relationships over 396 days. Figure 1a, left depicts the 2D embedding of the 42 million users, and details of the clusters are listed in Supplementary Table 1. There is no clear evidence of political conflicts in these clusters composed of millions of users. We conducted recursive clustering and detected two highly politically polarised subclusters (visualised in Fig. 1a, right) and others that were not (details are presented in Supplementary Table 2). Table 1 presents the basic information of both ECs. We found that most of the top TF-IDF words of the two subclusters (11.02 (left) and 11.01 (right)) were related to politics; the former represents criticism of the current conservative parties and Prime Minister Abe, and the latter is critical of the liberal party, Korea, China, and North Korea. The top 100 retweeted/replied users of each subcluster include famous polarised influencers or liberal (Mainichi) or conservative (Sankei) news media. Thus, we analysed the two subclusters as political ECs in comparison with other subclusters. Hereinafter, we refer to a subcluster as a community.

**Table 1:** Details of the left- and right-leaning ECs.

	Left EC	Right EC
#Users	251,036	269,791
#Retweets/replies	13,371,458	9,266,349
Top TF-IDF words (Translated to English)	Japan, Abe, National, Liberal Democratic Party, Abe administration, Member of Parliament, Prime Minister Abe, Problem,	South Korea, Japan, Member of parliament, China, Japanese, People, Opposition party, North Korea, Issue, Internet, Communist party,
Top influencers	Kikko_no_blog, knife9000, Dgoutokuji, KazuhiroSoda, TomoMachi, nogutiya, ISOKO_MOCHIZUKI, shiikazuo, wanpakuten, Beriozka1917	Katsuyatakasu, hyakutanaoki, Sankei_news, anonymous201504, KadotaRyusho, takenoma, smith796000, arimoto_kaori, SatoMasahisa, dappi2019,

Both ECs were highly independent of other communities: 72.6% (left) and 74.2% (right) of the original tweets of the retweets/replies were posted in the same EC (Fig. 1b), despite its small fraction of users in comparison with the 42 million total users. The second most frequent information source for the users of both ECs was the “01.01 General news” cluster (8.5%(left) and 8.1%(right)). This cluster was mostly composed of the general news media and people who followed them. The users of an EC were considered to spread the general news to their EC with a political bias. The third most frequent information source for both ECs was the opposite EC (2.1% (left) and 3.4% (right)). In other words, these clusters were independent but shared the same interests and interacted with each other.

**Figure 2:**
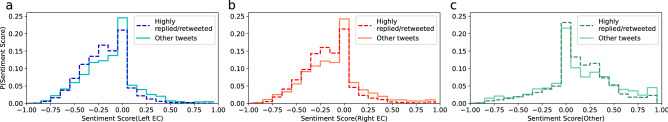
Sentiment score of tweets in left-(**a**) and right-leaning ECs (**b**) and other communities (**c**). The distribution of the sentiment score of highly replied/retweeted tweets (replied/retweeted more than 1000 times) and other tweets are plotted in each figure. The sentiment score of a tweet is calculated using the Google Natural Language API^52^ for 3,000 sampled tweets of each group.

Figure 1c depicts the daily internal information ratio (the ratio that the origin of a reply/retweet from the community is the same community). The ratio for both ECs was consistently high except for the sharp drop in October, when a major typhoon occurred in Japan causing extensive damage. However, despite the continued typhoon damage, the decline in the internal information ratio recovered within two days. In other words, user engagement with the EC was resilient even with exogenous forces. We also observed some peaks in the internal information ratio: The most significant peak for the left EC was the “Sakura-viewing party”, which was a spring party of supporters of Prime Minister Abe using public money. The controversial art exhibition of Aichi Triennale arose in the right EC; the artwork was considered an appeal for peace (left EC) or an insult to Emperor Hirohito (right EC). After the initial excitement, the users of each EC seemed not to have tired of it, and continued the engagement. Moreover, the tweets spread in each EC demonstrated a significantly higher negative sentiment than that of other tweets. Figure 2 shows the sentiment of highly replied/retweeted tweets and other tweets in left-(a) and right-leaning ECs(b) and other communities(c), using the Google Natural Language API^52^. Highly replied/retweeted tweets were tweets that were replied or retweeted more than 1000 times (100 times in 10% sampling). Figure 2a,b shows that the sentiment score of the tweets in both ECs leans toward the negative sentiment in contrast to the positive sentiment of other tweets (Fig. 2c). We also found that highly replied/retweeted tweets of both ECs leaned more toward the negative sentiment than other tweets. Thus, the ECs are fulfilled by the negative atmosphere, and highly spread information is more likely to have a negative sentiment. These analyses confirm the existence of continuously independent ECs composed of several hundred thousand users in the Japanese Twitter, where negative and political biased information is spread.

### Dense core-periphery structure of political echo chambers

**Figure 3:**
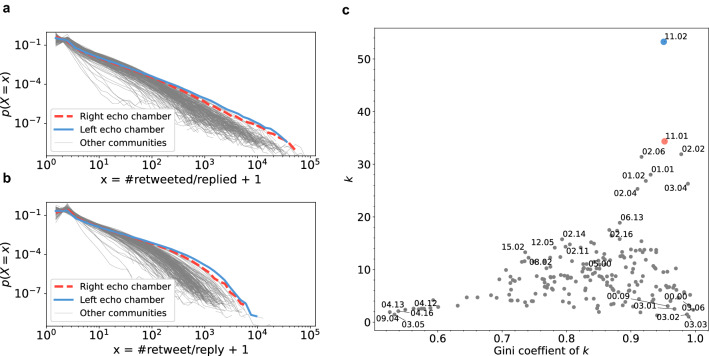
Dense core structure of a political EC: (**a**) In-degree (the number of retweeted/replied users) distribution of the left and right ECs with other communities. (**b**) Out-degree (the number of reply/retweet users) distribution of the left and right ECs with other communities. (**c**) Scatter plot of each community. The horizontal axis indicates the Gini indicator of degree distributions and the vertical axis indicates the average number of neighbours.

The in-degree distribution (number of neighbours who reply or retweet) of the two EC communities follows a scale-free distribution (Fig. 3a). The slope $\alpha$ of the in-degree distributions of the two communities exhibits low values (1.94 and 2.00). In other words, information sources are concentrated on specific core users, and peripheral users have little influence. The heterogeneous out-degree distribution of the network indicates efficient information spreading^45,53^. Figure 3a indicates that the slope of the degree distribution of both ECs is gentler than that of most other communities. We evaluated the degree concentration using the Gini indicator of degree, because some communities do not have a scale-free out-degree distribution. The horizontal axis of the scatter plot of Fig. 3b shows the out-degree of users. The Gini indicator also indicates that the information source of the two ECs is concentrated on a few users in comparison with most other communities.

We also found that the average degree of reply/retweet networks of both ECs is significantly higher than that in other communities. As shown in Fig. 3c, the average degrees of the left- and right-leaning ECs are 32.0 and 53.0, respectively. Given that the analysed data represents 10% of the randomly sampled date from the entire dataset, the detected neighbours are likely to be frequent reply/retweet sources for the user, and there are several missing relationships. Figure 3c shows that a larger percentage of users in both ECs frequently reply/retweet in comparison with that in other communities. We observed a moderate correlation between the number of retweets/replies and replied/retweeted (Supplementary Fig. 1). This indicates that influential users are more actively engaged in the community. According to these analyses, both ECs have efficient information-spreading networks with a dense and core-periphery structure, and the enthusiastic core nodes have essential roles in information spreading.

The various types of communities are depicted in Fig. 3c: “dense with strong core (top right)”, “star topology (bottom right)”, “loose community (bottom left)”, and “intermediate type (middle)”. Both ECs are categorised as “dense with strong core”, along with some other communities. 02.06 and 02.02 represent pop-star communities composed of highly influential pop-star/game accounts that feature pop stars/illustrators and enthusiastic fans. 01.01 represents the general news community, with several famous news media accounts (e.g., Yahoo News Topics). Community 03.04 represents the group of campaigns or prize competitions, whose users enthusiastically participate in multiple gift campaigns, such as “retweet and win the lottery”. ECs have denser information-spreading networks than these highly addictive communities. There are also “Star topology” (sparse core-periphery) communities, such as 00.00, represent Korean pop stars and the fans who mainly follow them. Another community type is the “loose community (bottom left)”, which does not have significant core nodes (bottom left of Fig. 3c). For example, communities 04.13 and 04.16 are local communities for teens and university students, where users meet and mingle online. “Intermediate type (middle)” communities are those that have relatively dense links and some moderate information spreading cores (i.e., 08.02: handcraft communities, 12.05: online games, and 15.02: LGBT communities). These comparisons signify the EC’s dense core-periphery structure in comparison with those of other communities.

### Connected core of political echo chamber

**Figure 4:**
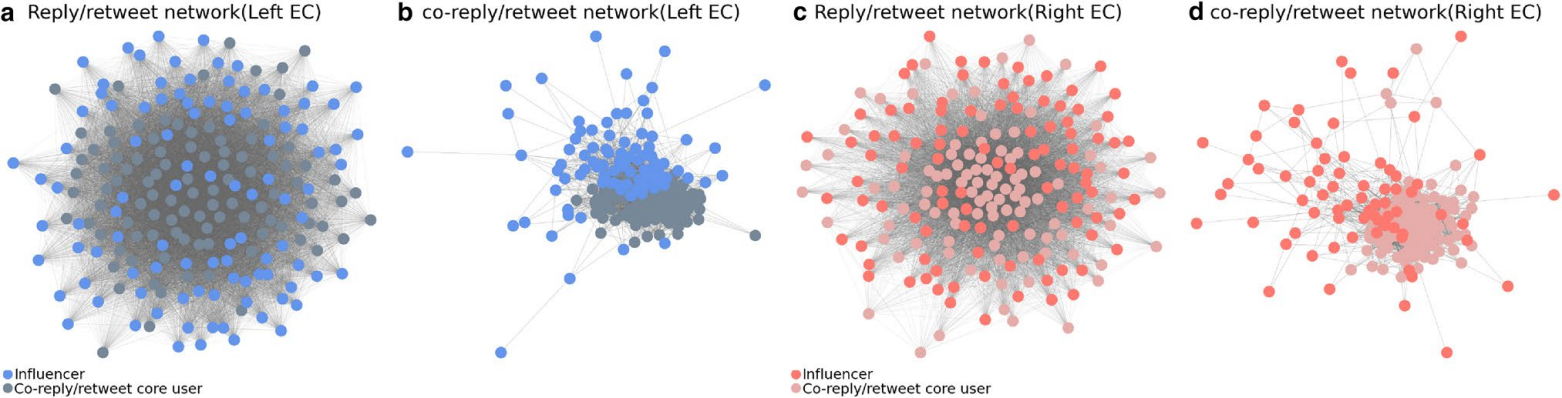
Network visualisation of the top 100 influencers (blue/red) and co-reply/retweet core users (light dark blue/dark red) of the EC communities: (**a**) Reply/retweet network of left EC. (**b**) The largest connected component of co-reply/retweet network of the left EC. 11 top influencer is not included in the component. (**c**) Reply/retweet network of right EC. (**d**) The largest connected component of co-reply/retweet network of the right EC. 30 top influencer are not included in the component.

The top influencers are tightly connected in information spreading networks. We confirmed 3,533 (left) and 2,002 (right) links between the top 100 influencers, and 150,907 (left) and 108,434 (right) links between the top 1000 influencers. The information densely disseminates between the top influencers. Considering the 10% sampling rate, the density of the actual network is several times larger than that observed. The force-spring layout of influencers in Fig. 4a,c also shows the dense information spreading network. Thus, the top influencers are not independent but resonate with each other. In both ECs, in addition to some independent core users, we do not observe significant clusters composed of several core users in Fig. 3a,c. The unilateral information spreading from the connected core may cause the homogeneity of opinions in each EC because influencers may hesitate to tweet different opinions in a densely connected network.

**Figure 5:**
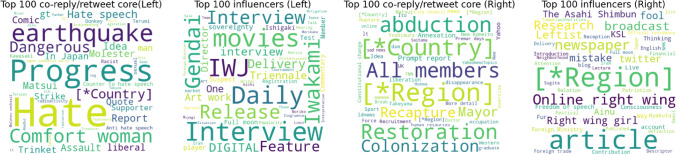
Word cloud^54^ visualisation of top frequently tweeted words of each group of the core users in each EC: The top 50 exclusively frequent words in the influencer/co-reply/retweet core for both groups are listed. The words are limited to words tweeted more than 30 times per day in each set of top 100 users. Personal information and country or place names are concealed for privacy reasons.

The top 100 influencers of both ECs are composed of famous persons, such as politicians, professors, and industrialists. Supplemental Table 3 shows that the top 100 influencers of both ECs are occasionally retweeted/replied from outside. This approach may help them avoid making extremely polarised tweets, thus concealing their real opinions. Assuming that users with the same opinion are connected with each other, the typical opinion of the EC may underlie the users who are densely bidirectionally connected to each other. We simply selected the top 100 users who have the most co-reply and co-retweet neighbours (they reply/retweet each other), from other than the top 100 influencers. We define these users as a co-reply/retweet core. The top 50 exclusively tweeted words in the top 100 co-reply/retweet core/top 100 influencers in each EC are listed in Fig. 5. We found that co-reply/retweet core users use aggressive and extremely polarised words, such as “Molotov cocktail” (left) and “Annexation, Colonisation” (right). In both ECs, these users’ influence tends to be limited to each EC, and the probability of that information reaching the opposite EC is approximately half of that from the top 100 influencers (Supplementary Table 3). Thus, while avoiding online flaming from the opposite EC and other communities, co-reply/retweet core users use relatively extreme words and influence the community’s top influential users.

The important findings are that the top influencers are tightly connected with the co-reply/retweet core in the information spreading network (Fig. 4a,c). The links from the top influencers to the co-reply/retweet core are 1,191 (left EC) and 746 (right EC). This indicates a certain amount of influence from the co-reply/retweet core on the top influencers. The co-reply/retweet network (Fig. 4b,d) also indicates a moderate amount of co-reply/retweet connection between these groups. Although the amount of information spread by the co-reply/retweet core is not significantly high, these users’ indirect influence on the EC via the top influencers is not negligible. Mining the representative opinions of the community is a highly difficult problem to solve. However, our investigation highlights the straightforward opinion of ECs and suggests the importance of monitoring these user accounts to prevent the spread of extreme opinions.

### Early collective resonance of core nodes and unilateral information spreading

**Figure 6:**
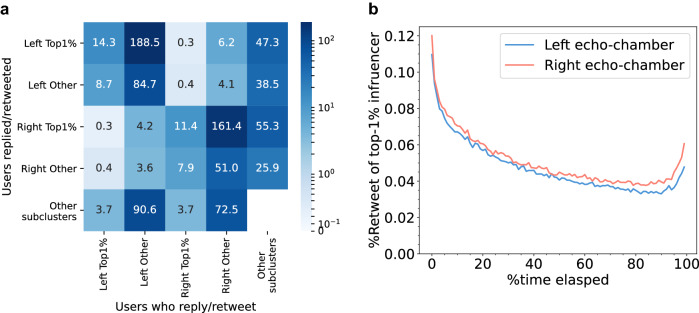
Role of influencers in information (reply/retweet) spreading in the left and right ECs: (**a**) The number (millions) of tweets transferred between groups according to the top 1% influencers/others of left EC/right EC and other subclusters. (**b**) Timeline of the average participation ratio of the top 1% influencers in the highly replied/retweeted tweets (retweeted/replied more than 1000 times) in each EC. The tweets arranged in the time series are divided into 100 equal groups, and the ratio is calculated for each period. We ignore the few tweets one week after the first reply/retweet.

Next, we investigated the information flow between influencers and other users in the left and right ECs. Figure 6a shows the amount of information spreading for the top 1% influencers and other users in each EC and other subclusters. We found that the top 1% influencers disseminate a large part of the information to other users in both ECs: 188.5 million (left)/161.4 million (right) retweets/replies. The amount of information (reply/retweet) is estimated from the 10% sample of tweets. In comparison with this result, the information transfer from the periphery to the influencer is considerably smaller: 8.7 million and 7.9 million, respectively. This result coincides with the estimation of the heterogeneous out-degree distribution of the network. Although peripheral users more significantly prefer the top 1% influencers as the target of retweet/reply, the major target of core users’ retweets/replies is in the ECs (62.2% in the right EC and 59.1% in left EC) despite a small proportion (1%) of users in each EC. In addition, not only are the top 1% influencers the targets of retweets and replies, but they also post a significant number of retweets and replies. The top 1% influencers account for 27.4 million (left) and 23.7 million (right) retweets and replies. The top 1% influencers of each EC post significantly frequent retweets and replies (27.1 (left) and 21.1 (right) per day). This result indicates that these influencers are highly engaged in spreading information and are a popular source of information spreading.

We also observed the early engagement of influencers in the information cascade. We selected the highly replied/retweeted tweets that were retweeted/replied more than 1000 times in each EC and observed the timeline of influencers’ engagement. Majority of the tweets in most of the information cascades were posted in a few days. We ignored the tweets after a week and divided the tweets into 100 groups by order of time. The percentage of top users in each period (Fig. 6b) shows that influencers tend to engage in the early stage of the information cascades in both ECs. 10.9% (left) and 12.0% (right) of people who reply/retweet to the highly replied/retweeted tweets are among the top 1% influencers in the early stage, but the ratio decreases by approximately one-third in the later periods. We note that the increase in the rate in the final periods is due to some influencers who are devoted to following up widely spread information after the initial burst settles.

In most cases, a user may have seen the original tweet through someone’s retweet before he/she replied or retweeted to the process. However, the recorded data of who replied or retweeted the original tweet and the detailed spreading process are unknown. From the early engagement of influencers and the high reply/retweet frequency between them, we suppose that the original tweet first resonates between influencers, and then the information spreads to the other users. The highly replied/retweeted tweets are likely to have a higher negative sentiment (Fig. 2a,b). Therefore, the influencers have much to contribute to make the community a negative atmosphere. These results indicate the typical process of information cascade in the EC: First, influencers or other users post a tweet, and enthusiastic influencers amplify the information, particularly a negative tweet; then, submissive users follow the information.

## Discussion

We extracted the left- and right-leaning ECs from all the Japanese users and long-term observations between the political ECs. The sizes of the ECs were nearly the same (251,000 on the left and 270,000 on the right), and the contents in both ECs were firmly polarised each day. With regard to the 521,000 users in the ECs, the EC is not overstated^16^: Users of each community always receive and send sharply polarised information. In other words, ordinary intensively polarised ECs exist and can be extracted using network community detection from all reply/retweet relationships. These observations are limited to Japan, which has the second-largest number of users. However, the tendency of the subjects discussed in both ECs is consistent with those of the United States^55^: The left EC discusses the current conservative party, and the right discusses foreign threats. The tweets in these communities have significantly negative sentiments in comparison with those of other communities.

This negative-sentiment information is spread efficiently and unilaterally by small influencers in both ECs. The core-periphery and core-connected structure exhibits viral and efficient information spreading in the EC. The core nodes are connected to each other; this result is consistent with those of previous studies^11,14^ that indicated the existence and importance of core users. We also found that core users tend to engage in the early states of information cascading. This analysis helps in understanding the individual case of information spreading. For example, previous studies indicate faster and deeper (long network diameter) spreading of fake news^8^. The sequential spreading from the core to the periphery leaves a footprint of the deep chain of information spreading. Our results may contribute to the improvement in the detection of hate speeches, online flaming, and aggressive behaviours.

We also found that the co-reply/retweet core includes co-replies/retweets that are firmly tied to “mild” influencers. Co-reply/retweet core users radically use offensive words and claim conspiracy theories. In terms of the amount of information spreading, the influencers represent these communities. However, the fundamental opinion may lie in the co-reply/retweet core tweets because the influencer, who is likely to be a famous person, posts “sophisticated” tweets that are not offensive and are politically correct. Moreover, the potential influence from the co-reply/retweet core to the influencer should not be ignored. Further research is needed to extract the real, hidden opinions of the community using network core detection^56,57^, which is optimised for opinion detection.

The limitations of this study are the clustering method and the data-collection period. We selected the most accurate clustering algorithm within the constraints of running the analysis in real time. The detected communities were well separated from each other from the extracted top keywords. Moreover, further discussion regarding the content of individual tweets and diffusion-based information in psychological and political science is required. It is unclear whether the same size, structure, and information spreading process of both political ECs are coincidental or deliberate. Thus, further analysis is required to determine how ECs maintain their efficient information spreading network by analysing long-term data.

## Data

We collected 10% of the randomly sampled Japanese tweets from the data supplier. The data included 2,491,216,473 tweets from 43,159,920 users from 1 December 2018 to 31 December 2019. The tweets included 633,794,720 replies and 843,605,425 retweets. We analysed the 42,132,385 users in the largest connected component of the reply/retweet network. The data for December 2019 contained very few tweets on COVID-19. Some of the collected tweets contained non-Japanese tweets. Users who tweeted this information were concentrated on some subclusters (the clustering method is described in the Methods section) and were manually removed. The removed subclusters were engaged in clusters 10 and 16, and subclusters 00.15, 07.03, 11.09, 15.00, 15.01, and 15.05.

## Methods

### Detection of political ECs and other communities

To extract the groups of users wherein information was densely dissimulated, we clustered the users using the Leiden clustering method^49^ on the basis of their reply/retweet relationships. Each edge of the network was weighted by the number of replies/retweets between the two users. This method searches for the best cluster set for which the modularity Q value^58^ is maximised. The Leiden method is widely used in academic communities owing to its high clustering accuracy and high processing speed in comparison with that of other methods^58,59^. Because some of the retrieved clusters contain millions of users, it is difficult to detect sharp conflicts between them. We recursively clusterised the clusters containing more than 500,000 users. For comparison with the community containing insufficient users, we did not recursively clusterise the clusters with 500,000 or lesser users. The retrieved (sub)clusters were named xx.yy, where xx represents the cluster number, and yy denotes the subcluster number. The subclusters with less than 50,000 users were grouped together named “xx.99”. Clusters that were not recursively clusterised were named “xx...”. We analysed the clusters/subclusters containing a certain number of users (more than 50,000).

### Extraction of representative terms of clusters and subclusters

We extracted the representative terms using TF-IDF^60^, which is a measure of the amount of information that the term has^61^ from the concatenated text of all the tweets of the clusters and subclusters. We extracted nouns, verbs, adverbs, and adjectives from the text using MeCab. Then, TF-IDF was calculated using the equation $\text {tfidf} (t, d) = \text {tf} (t,d) \cdot \text {idf}(t)$, where t and d represent the term and the concatenated texts, respectively, $\text {tf}(t, d) = \log {(1 + f_{t, d})}$ is the log value of the frequency of the term *t* in document *d*, and $\text {idf}(t) = \log \frac{N}{df_{t}}$ is the inverse ratio of the number of concatenated texts that include the term $df_{t}$.

We used the ratio of the term frequency in the co-reply/retweet core/influencers to that in both to extract the representative terms in both groups. The frequency was calculated independently in each EC.

### Extraction of network influencers and co-reply/retweet core of subclusters

The k-core^56^ and other recent methods^57^ have been proposed for the core detection of a community. However, for the sake of simplistic understanding, we used a simple ranking to determine the top authorship. The top-N influencers represent the top-N most retweeted/replied users. The top-N co-reply/retweet users are the top-N users in the ranking of the number of bidirectional links the user has.

### Network visualisation

The reply/retweet network composed of 42 million nodes was visualised using network representation learning (LINE^50^) and the dimension reduction method (UMAP^51^). LINE creates a map of nodes in a high-dimensional (128-dimensional) space, preserving the local structure. Intuitively, the connected nodes are located close to each other, whereas other node pairs are not. UMAP is a state-the-art method of dimension reduction that maps the global structure while preserving the local structure. The network between influencers and the co-reply/retweet core (the top users who have the most co-reply/retweet neighbours) was visualised using the force-spring layout, which is a common layout that provides an intuitive map.

## Supplementary information

Supplementary Information

## Data Availability

The full datasets used in this study are not publicly available, but are available from the corresponding author on reasonable request. Most of them can be retrieved from Twitter.com, except for the deleted tweets.
