# Supplementary material for: Dense and influential core promotion of daily viral information spread in political echo chambers
Source: Sci Rep. 2021 Apr 5;11:7491. doi: 10.1038/s41598-021-86750-w (PMC8021571; doi:10.1038/s41598-021-86750-w)
Supplement: Supplementary file 1 — Supplementary Information [file 41598_2021_86750_MOESM1_ESM.pdf]

Dense and influential core promotes daily viral information spreading in political echo chambers

Kimitaka Asatani, Hiroko Yamano, Takeshi Sakaki, Ichiro Sakata

Supplementary Table S1: List of clusters

The following table shows the list of retrieved clusters by network clustering (Leiden clustering method) from 42 million users' reply-retweet relationships. The authors posteriorly determined the cluster name of each cluster. We retrieved clusters that contained more than 50,000 users. The clusters were numbered from 0 by order of the number of users. The top TF-IDF words were Japanese (shown in the bottom part of each block) and were translated to English (shown in second from the bottom of each block). We used Google Translate, and then humans modified some parts of the results. Cluster 10 is an English-speaking persons' cluster, and 16 is Chinese. We excluded these clusters from the analyses.

#Cluster	Name	#Users	#Links
Top influencer account			
Top TF-IDF words(Translated to English)			
Top TF-IDF words			
0	Korean pop star(Kpop)	5,101,483	39,897,359
NCT_OFFICIAL_JP, BTS_jp_official, Kstyle_news, WayV_official, BTS_twt, official_izone, TastyJapan, NCTsm..			
JJ, bts, twice, seventeen, izone, exo, nct127, nct, got7, japan, in, ikon, the, Aizuwan, Yuchun, Dong Ba..			
ジェジュン, bts, twice, seventeen, izone, exo, nct127, nct, got7, japan, i..			
1	News / hobby	4,054,673	115,593,741
livedoornews, YahooNewsTopics, kyono_iyashi, minnano_dougaww, tyomateee, nhk_news, yeskiri, HEISEI_love_..			
Love, Japan, feel, work, man, teacher, photography, cinema, public, cartoon, best, work, game, please, w..			
好き, 日本, 感じ, 仕事, 人間, 先生, 写真, 映画, 公開, 漫画, 最高, 作品, ゲーム, お願い, 世界, 子供, 開催,..			
2	Game for male	3,714,056	94,215,831
bang_dream_gbp, imascg_stage, fgoproject, granbluefantasy, 33kitta, purinharumaki, azurlane_staff, bang_..			
Love, videos, campaigns, delivery, gift, fgo, characters, the day before ratio, cartoon, events, forums,..			
好き, 動画, キャンペーン, 配信, プレゼント, fgo, キャラ, 前日比, 漫画, イベント, 質問箱, 公開, アニメ, バンド..			
3	Campaign	3,574,115	25,715,847
akiko_lawson, yousuck2020, NitoriOfficial, McDonaldsJapan, Kirin_Company, AEON_JAPAN, GalaxyMobileJP, JA..			
Campaign, gifts, lottery, applicants can join, post, How to Apply, coupon, retweets, amp, challenge, opp..			
キャンペーン, プレゼント, 抽選, 応募, 参加, 投稿, 応募方法, クーポン, リツイート, amp, チャレンジ, チャンス, 動..			
4	Teenagers/student	2,810,481	14,889,118
bozu_108, hachi_08, hajimesyacho, hikakin, Popteen_cgw, sankoiti_offici, Yahoo_GYAO, kantamizutamari, ud..			
Forum, peing, like, question, EmiEmi, videos, best, fun, gifts, please, band, photo, public, ticket, tou..			
質問箱, peing, 好き, 質問, 笑笑, 動画, 最高, 楽しみ, プレゼント, お願い, バンド, 写真, 公開, チケット, ツ..			
5	Offline hobby/etc	2,744,274	31,360,042
345___chan, love2chiitan, neko2time, aveirjapan, pamyurin, otakunews6398, mofunekoc1, ehara_hiroyuki_, Sa..			
'Delivering, mutual follow-up, please, photo, love, video, distribution, followers, cheer, fun, work, acc..			
配信中, 相互フォロー, お願い, 写真, 好き, 動画, 配信, フォロワー, 応援, 楽しみ, 仕事, アカウント, イベント, am..			
6	Male pop tar	2,543,124	32,905,899
oricon, modelpress, natalie_mu, AbemaTV, fns_kayousai, thetvjp, arashi5official, eiga_natalie, Mst_com, ..			
Kisumai, love, photo, fan, movie, fun, sixtones, news, broadcast, performers, best, love, Johnny, amp, s..			
キスマイ, 好き, 写真, ファン, 映画, 楽しみ, sixtones, news, 放送, 出演, 最高, 大好き, ジャニーズ, a..			
7	Sexual	2,221,238	12,121,460
av_tamashii, lov_boscat, jg86d, jinghuahuicui, Meutann15, kein_yarisugi, adaruto30douga, Love_PersonNo1,..			
Saffle, recruiting, erotic, girls, video, Ofupako, line, etch, Masturbation, Ona, dm, Prof, virgin, Foru..			
セフレ, 募集, エロ, 女子, 動画, オフパコ, line, エッチ, オナニー, オナ, dm, プロフ, 童貞, 質問箱, 好き,..			
8	Game for female	2,209,473	30,497,230
kimetsu_off, avogado6, hypnosismic, hanae0626, ensemble_stars, iD7Mng_Ogami, KAJI_staff, ichibanKUJI, co..			
Exchange, please, transfer, like, the public, can badge, events, gifts, fun, mail, possible, rude, illus..			
交換, お願い, 譲渡, 好き, 公開, 缶バッジ, イベント, プレゼント, 楽しみ, 郵送, 可能, 失礼, イラスト, 誕生日, 手..			

<table>
<tr><td>9</td><td>Sports/etc</td><td>1,985,822</td><td>11,475,011</td></tr>
<tr><td colspan="4">omosiro_geki, Twitube_123, kokoro_odoru_1, omosirooidouga, YutoNagatomo5, kusowaraeruwwww, joker_budou, ..</td></tr>
<tr><td colspan="4">Players, game, Forum, peing, team, cheering, pitcher, coach, questions, vs, soccer, gifts, Giants, Hansh..</td></tr>
<tr><td colspan="4">選手, 試合, 質問箱, peing, チーム, 応援, 投手, 監督, 質問, vs, サッカー, プレゼント, 巨人, 阪神, 優勝,..</td></tr>
<tr><td>10</td><td>-</td><td>1,953,911</td><td>2,641,865</td></tr>
<tr><td colspan="4">9GAG, ArianaGrande, ogecebel, yousannnn, bunnyarchive, Jerrypleasure, NihongoSOS, sincerojesuis, carIys,..</td></tr>
<tr><td colspan="4">-..</td></tr>
<tr><td colspan="4">the, de, me, you, my, and, que, no, ゲスト, is, in, of, this, la, it, ゜゜..</td></tr>
<tr><td>11</td><td>News/politics/etc</td><td>1,778,741</td><td>35,252,247</td></tr>
<tr><td colspan="4">Sankei_news, konotarogomame, chowtingagnes, katsuyatakasu, mainichi, rugbyworldcupjp, AbeShinzo, nikkei,..</td></tr>
<tr><td colspan="4">Japan, Korea, people, problem, lawmakers, the Liberal Democratic Party, news, Abe, Japanese, Chinese, op..</td></tr>
<tr><td colspan="4">日本, 韓国, 国民, 問題, 議員, 自民党, ニュース, 安倍, 日本人, 中国, 野党, 批判, 安倍首相, nhk, 政府, 安倍..</td></tr>
<tr><td>12</td><td>Online Game</td><td>1,685,770</td><td>17,773,812</td></tr>
<tr><td colspan="4">SplatoonJP, FortniteJP, ara_to1, FF_XIV_JP, ApexLegendsWiki, Otojya, umadori0726, EAA_tw, VodkaChaso, ___..</td></tr>
<tr><td colspan="4">Ps4, Forum, Fort Night, peing, game, switch, Splatoon 2, nintendo, question, ff14, splatoon2, please, sh..</td></tr>
<tr><td colspan="4">ps4, 質問箱, フォートナイト, peing, ゲーム, switch, スプラトゥーン 2, nintendo, 質問, ff14, ..</td></tr>
<tr><td>13</td><td>Game streaming/offline</td><td>1,449,051</td><td>17,051,368</td></tr>
<tr><td colspan="4">Jel___official, uni_mafumafu, StPri_info, p_ma_ru, uratasama, satomimi___, _amatsuki_, Colon56Nsab, soraru..</td></tr>
<tr><td colspan="4">Strike, pre, www, videos, forums, like, post, peing, fun, colon, ww, Mafumafu, 莉犬, delivery, goods, trie..</td></tr>
<tr><td colspan="4">すと, ぷり, www, 動画, 質問箱, 好き, 投稿, peing, 楽しみ, ころん, ww, まふまふ, 莉犬, 配信, グッズ,..</td></tr>
<tr><td>14</td><td>Mobile FPS game</td><td>1,163,593</td><td>12,974,199</td></tr>
<tr><td colspan="4">GAME_KNIVES_OUT, tyoumukakin7, yuukoru_, hitohito12O, QOQO114514114, oreratuyoi, mirrativ_jp, ginnan_0, ..</td></tr>
<tr><td colspan="4">Wilderness action, guerrilla, participation, wilderness, determined, Forum, conditions, please, duo, pri..</td></tr>
<tr><td colspan="4">荒野行動, ゲリラ, 参加, 荒野, 確定, 質問箱, 条件, お願い, デュオ, 賞金, キル, リプ, peing, 配信, クラン,..</td></tr>
<tr><td>15</td><td>LGBT</td><td>634,617</td><td>4,764,726</td></tr>
<tr><td colspan="4">williejia, THECUMCONTROL, LanNick8, hyaku1063, chenhui_hifunbb, Dubeyyjun, gayfetishjp, THEMUSCLEMANS, t..</td></tr>
<tr><td colspan="4">☒☒, personal communication, ☒逼, self, ☒巴, 无套, Forum, gay, ☒个, resources, 哈哈, bicine, peing, love, video,..</td></tr>
<tr><td colspan="4">☒☒, 私信, ☒逼, 自己, ☒巴, 无套, 質問箱, ゲイ, ☒个, ☒源, 哈哈, 微信, peing, 好き, 動画, 需要, ☒..</td></tr>
<tr><td>16</td><td>-</td><td>583,765</td><td>3,708,427</td></tr>
<tr><td colspan="4">VOAChinese, RFA_Chinese, lihkg_forum, bbcchinese, TuCaoFakeNews, IntyPython, nytchinese, LifetimeUSCN, X..</td></tr>
<tr><td colspan="4">-..</td></tr>
<tr><td colspan="4">香港, 中国, 美国, 中共, 自己, 什么, 如果, 人民, ☒个, 政府, 警察, 中國, 国家, ☒在, 民主, 你☒, 香港人, ..</td></tr>
<tr><td>17</td><td>"NGT46"</td><td>413,515</td><td>5,202,410</td></tr>
<tr><td colspan="4">nogizaka46, keyakizaka46, nogisatsu, otome_kagura, nogikoiofficial, mechakari, hinatazaka46, yuuui_imaiz..</td></tr>
<tr><td colspan="4">Nogizaka 46, zelkova slope 46, love Nogi, corps, the sun slope 46, Nogizaka, EmiEmi, id, spontaneous, Na..</td></tr>
<tr><td colspan="4">乃木坂 46, 欅坂 46, 乃木恋, 軍団, 日向坂 46, 乃木坂, 笑笑, id, 自発, 西野七瀬, 乃木, メンバー, 写真集, 齋藤..</td></tr>
<tr><td>18</td><td>"Identity "</td><td>411,287</td><td>4,711,302</td></tr>
<tr><td colspan="4">IdentityVJP, IdentityV_info, 5tukirin, Rcollection_PR, MotimotiPopo, nyulouis, wolf5rin, identityV_stage..</td></tr>
<tr><td colspan="4">Personality, identityv, hunter, illustration, love, Forum, applicants, peing, run, costumes, Shinoarisu,..</td></tr>
<tr><td colspan="4">人格, identityv, ハンター, イラスト, 好き, 質問箱, 応募, peing, ラン, 衣装, シノアリス, プレゼント, ..</td></tr>
<tr><td>19</td><td>figure skating</td><td>266,702</td><td>3,316,463</td></tr>
<tr><td colspan="4">YoshikiOfficial, HydeOfficial_, asahi_photo, KinbakuTw, mainichiphoto, SUGIZOofficial, sn_figure, Toshlo..</td></tr>
<tr><td colspan="4">Hanyu, players, Yuzuru Hanyu, figure skating, hyde, yoshiki, AkiraMigaku, photos, fan, Yuitsuru, perform..</td></tr>
<tr><td colspan="4">羽生, 選手, 羽生結弦, フィギュアスケート, hyde, yoshiki, 昌磨, 写真, ファン, 結弦, 公演, 演技, 高橋大輔..</td></tr>
<tr><td>20</td><td>"Dragon Ball Legends"</td><td>163,273</td><td>1,276,171</td></tr>
<tr><td colspan="4">Nenaro_YouTube, iiwaneOBASANwww, Siitake_Oisii, noa_dbh, mnlselvvigets5, amour_macherie, hentaiblackcat,..</td></tr>
<tr><td colspan="4">Narikiri, bot, r1, love, fufu, okay, pick-up, reaction, Forum, partner, lady, please, question, pickpock..</td></tr>
<tr><td colspan="4">なりきり, bot, r1, 好き, ふふ, 大丈夫, お迎え, 反応, 質問箱, 相手, 貴女, お願い, 質問, すり, きっと, p..</td></tr>
<tr><td>21</td><td>"Pokemon(Go)"</td><td>134,001</td><td>1,410,747</td></tr>
<tr><td colspan="4">PokemonGOAppJP, gamewith_pkgo, pokemongo_db, DFF_OperaOmnia, ekimemo, YAMADA_N_DoMo, nian..</td></tr>
<tr><td colspan="4">Pokemon go, different colors, raid, Pokemon, Romancing SaGa, characters, events, forums, station memo, w..</td></tr>
<tr><td colspan="4">ポケモン go, 色違い, レイド, ポケモン, ロマサガ, キャラ, イベント, 質問箱, 駅メモ, ww, プレゼント, go, ポケ,..</td></tr>
<tr><td>22</td><td>Online Radio</td><td>68,672</td><td>549,142</td></tr>
<tr><td colspan="4">spoon_radio, sushizanmaikabu, Tomato556931, Shika_Barten, bacon_kirai, 294_virtual, cana1a7acan, akaneir..</td></tr>
</table>

Forum, peing, delivery, question, spoon, like, EmiEmi, reality, anonymous, ww, in recruiting, okay, lt, ..			
質問箱, peing, 配信, 質問, spoon, 好き, 笑笑, reality, 匿名, ww, 募集中, 大丈夫, lt, 仕事,..			
23	Community of teenage girls	54,371	240,738
Rabi________oO, mm_skm9, MiuPima, 6ai5ueO, O27NO, nynkfktm, DtyVi, l93Ott, typhoon_____y, Y1fOe, pxxx1_dxk, P..			
Information, please, under the umbrella, planning, roses, nucleic acid, cooperation, standards, free of ..			
情報, お願い, 傘下, 企画, ばら, 核酸, 協力, 規格, 無償, dm, 初心者, 失礼, 酸化, 当方, ho, 出戻り, スパ..			

Supplementary Table S2: List of sub-clusters

The following long table shows the list of retrieved sub-clusters by recursive network clustering. The clusters that contained more than 500,000 users were divided into subclusters. We retrieved the subclusters that contained more than 50,000 users, and named them xx.yy (xx: cluster number, yy: subcluster number). The subclusters were numbered in order of the number of its users. Other small clusters were gathered to one subcluster named xx.99. The authors posteriorly determined the cluster name of each subcluster from the top TF-IDF words and top influencers. Some of the collected tweets contain non-Japanese tweets. Users who made these tweets are concentrated on some subclusters (the clustering method is described in the Methods section) and were manually removed. The removed subclusters were engaged in clusters 10 and 16, and subclusters 00.15, 07.03, 11.09, 15.00, 15.01, and 15.05.

From the results of the top authors and top TF-IDF words, subcluster 11.02 is the left EC, and 11.01 is the right EC. There is no significant evidence of political polarization in other clusters.

#Cluster	Name	#Users	#Links	α of the scale free distribution	#Links between top 100 influencers
Top influencer account					
Top TF-IDF words(Translated to English)					
Top TF-IDF words					
00.00	Kpop - ”BTS”	710,925	2,285,013	1.816	273
’BTS_jp_official’, ’Billboard_JAPAN’, ’Smeraldo_Books’, ’BigHitEnt’, ’uruhiko_kpop’, ’BT21_Japan’, ’lott..					
Bts, jimin, taehyung, jungkook, jin, bt, Tete, Ji Min, bulletproof Boy Scouts, btsv, elf, japan, gin, th..					
bts, jimin, taehyung, jungkook, jin, bt, テテ, ジミン, 防弾少年団, btsv, elf, j..					
00.01	Kpop - ”NCT”	609,035	2,820,130	2.076	415
’NCT_OFFICIAL_JP’, ’WayV_official’, ’NCTsmtown’, ’NCTsmtown_DREAM’, ’pianissimo_jh’, ’vivi_magazine’, ’n..					
Nct, nct127, wayv, Wei God, weishenv, jaehyun, Jae-hyun, winwin, nctdream, japan, mark, weibo, Do Young,..					
nct, nct127, wayv, 威神, weishenv, jaehyun, ジェヒョン, winwin, nctdream, ja..					
00.02	Kpop - ”Kang Daniel”	451,196	2,948,212	1.909	129
’WannaOne_twt’, ’x1fancafe’, ’pdx101_rr’, ’RE_ONG950825’, ’MnetMcountdown’, ’kconjapan’, ’LunarPlanet121..					
Kangdaniel, x1, cans Daniel, Hason’un, fluvial, hasungwoon, wannaone, ongseongwu, sungwoon, Onson’u, ab6..					
kangdaniel, x1, カンダニエル, ハソンウン, 河成, hasungwoon, wannaone, ongseongwu, ..					
00.03	Kpop - ”BTS2”	422,361	5,004,288	2.062	678
’BTS_twt’, ’bangtan_lab’, ’BTS_hope_jk’, ’bts_bighit’, ’jimchim_bts’, ’bts123_bantan’, ’BTSHtogether’, ’..					
Bts, Ji Min, army, Tete, kuk, Jin, Yoon, Jong Kook, Vantan, Tae-hyun, jimin, bulletproof Boy Scouts, Nam..					
bts, ジミン, army, テテ, グク, ジン, ユンギ, ジョングク, バンタン, テヒョン, jimin, 防弾少年団, ナム,..					
00.04	Kpop - ”IZONE”	321,266	2,115,758	1.982	286
’official_izone’, ’keitadj’, ’GFRDofficialJP’, ’MnetKR’, ’39_kirschesaku’, ’RVsmtown’, ’IZONE_DAILY’, ’I..					
Izone, Aizuwan, iz, one, gfriend, SakiRyo, Miyawaki, Wonyon, miyawakisakura, Kimuminju, kimminju, Chewon..					
izone, アイズワン, iz, one, gfriend, 咲良, 宮脇, ウォニョン, miyawakisakura, キムミンジュ..					
00.05	Kpop - ”EXO”	320,680	1,778,538	1.806	571
’Kstyle_news’, ’weareoneEXO’, ’chanyeolit’, ’EXO_NEWS_JP’, ’knockknock0408’, ’AppleMusicJapan’, ’Gloriou..					
Exo, chanyeol, Chanyoru, ☒烈, weareoneexo, kai, baekhyun, sehun, whatalife, sc, Suho, suho, Cefn, soo, Ka..					
exo, chanyeol, チャニョル, ☒烈, weareoneexo, kai, baekhyun, sehun, whatalif..					
00.06	Kpop - ”SEVENTEEN”	247,224	2,638,699	2.026	408
’abema_kpopdrama’, ’pledis_17jp’, ’pledis_17’, ’woozister’, ’raffine_svtww’, ’svt13_hd’, ’0a0k0o’, ’suki..					
Seventeen, Sebuchi, Wonu, Hoshi, Mingyu, carat, wonwoo, maggots, hoshi, Dogyomu, mingyu, Joshua, woozi, ..					
seventeen, セブチ, ウォヌ, ホシ, ミンギュ, carat, wonwoo, ウジ, hoshi, ドギョム, mingyu..					
00.07	Kpop - ”ikon”	244,581	1,497,429	1.897	353

'yuri_haru9', 'excite_music', 'YGJAPANofficial', 'nallalisa0327', 'wwd_jp', 'ohboy_27', 'furuyamasayuki0..				
Ikon, blackpink, lisa, Geneva, Hanbin, the second son, june, bobby, jinhwan, Babi, Lisa, winner, junhoe,..				
ikon, blackpink, lisa, ジュネ, ハンビン, ジナン, june, bobby, jinhwan, バビ, リサ, ..				
00.08	Kpop - "Twice"	200,432	1,904,189	2.035	382
'nhk_oto', 'JYPETWICE_JAPAN', 'MinatozakiJp29', 'twice55f', 'JYPETWICE', 'warnermusic_jp', 'TWICE_MM1020..				
Twice, Tsuwi, once, tzuyu, Sana, Mina, Twice, yong, Na-young, Dahyon, sana, peach, mina, nayeon, Ji Hyo,..				
twice, ツウィ, once, tzuyu, サナ, ミナ, トゥワイス, ジョンヨン, ナヨン, ダヒョン, sana, モモ, m..				
00.09	Kpop - "Shinwa"	195,571	595,285	2.403	497
'TastyJapan', 'Ohayoasa_', 'yuki7979seoul', 'Tastemade_japan', 'xxroamxx', 'osu4koyani', 'utaaaa1213', '..				
Picrew, tastyjapan, lucky, gt, lt, [illegible], manufacturers, [illegible], tv, Korea, shinhwa, recipes, sbs, 10, libre,..				
picrew, tastyjapan, lucky, gt, lt, [illegible], メーカー, [illegible], tv, 韓国, shinhwa, レ..				
00.10	Kpop - "BamBam"	178,813	1,019,722	1.966	641
'GOT7_Japan', 'TOWER_Shibuya', 'GOT7Official', 'sweetie_bam', 'JacksonWang852', 'mtuan93', 'doitnow_93',..				
Got7, mark, 2pm, bambam, jus, Juno, loop, jb, Jin Young, junho, jinyoung, mark, Tae Kyung, focus, yugyeo..				
got7, mark, 2pm, bambam, jus, ジュノ, loop, jb, ジニョン, junho, jinyoung, マ..				
00.11	Kpop - "UNIQ"	176,077	1,224,826	2.431	310
'XiaoZhan1005_TH', 'Zhangaiyi', 'Yibo_Thailand', 'BoJunYiXiao_TH', '3gepingguo', 'selaseanyw', 'Lunalypi..				
Cr, xiaozhan, logo, ish, saint, perthsaintsation, wangyibo, 2w, sup, you, OKazuhiro, weibo, mage, xiaozh..				
cr, xiaozhan, logo, ish, saint, perthsaintsation, wangyibo, 2w, sup, ..				
00.12	Kpop - "TVXQ"	151,071	2,006,206	1.916	808
'kor_celebrities', 'shinetter', 'shfly3424', 'SJ_NEWS_JP', 'AllRiseSilver', 'SHINee', 'kin_718', 'wow_ko..				
Juno, Dong Bang Shin Ki, Temin, taemin, Yunho, Changmin, shinee, yunho, tvxq, super, xv, 15t, junior, co..				
ユノ, 東方神起, テミン, taemin, ユンホ, チャンミン, shinee, yunho, tvxq, super, xv, 15..				
00.13	Kpop - "MONSTA_X"	140,601	937,543	2.041	492
'Official_MX_jp', 'OfficialMonstaX', 'pekepon_kpop', 'ATEEZofficialjp', 'MercuryTokyo_UM', 'Mnet_Japan',..				
Monsta, monstax, Wono, Minyoku, wonho, minhyuk, Shonu, Hyun won, shownu, ateez, hyungwon, Kihyon, alliga..				
monsta, monstax, ウォノ, ミニョク, wonho, minhyuk, ショヌ, ヒョンウォン, shownu, atee..				
00.14	Kpop - "JBJ95"	120,474	727,358	2.215	259
'Kkorepo', 'official_ONEUS', 'haruhana_tvg', 'news_pia', 'KpopStarzJP', 'ONEUS_JPN', 'RealVIXX_Japan', '..				
Jbj95, b1a4, oneus, vixx, Kim, Kenta, kenta, Hyun Joong, snuper, ace, onf, japan, supernova, Don, Sangyu..				
jbj95, b1a4, oneus, vixx, キム, ケンタ, kenta, ヒョンジュン, snuper, ace, onf, j..				
00.15	-	111,741	144,866	2.079	27
'idwiki', 'askmenfess', 'ipandacom', 'seterahdeh', 'bayu_joo', 'mahasiswikupu2', 'AlamiSadHomie', 'seren..				
-..				
aku, yang, mau, ada, ini, sama, di, you, aja, ya, thread, kamu, dan, ..				
00.16	Kpop - "straykids"	95,125	409,910	2.210	205
'Stray_Kids_JP', 'springginsour', '2000320kr', 'WE_THE_BOYZ', 'pp_hhj', 'THEBOYZJAPAN', 'visualglowhj', ..				
Straykids, theboyz, Sukizu, jin, hyunjin, miroh, stray, stay, hq, boyz, changbin, the, seungmin, felix, ..				
straykids, theboyz, スキズ, ヒョンジン, hyunjin, miroh, stray, stay, hq, boyz..				
00.17	Kpop - General	80,111	334,374	3.625	1,136
'Nieli1210', 'suga9339b', 'JIN_ARMY_____', 'bts_cy27_bh067', 'cm_____btsxx___bot', 'Jung_HoSeok_218', 'dolphi..				
Hyun, bot, pick-up, brother, Nuna, pen, sight, Hea, pick-up, love, fufu, room, kk, your room, okay, Oppa..				
ヒョン, bot, 出迎え, 兄さん, ヌナ, ペン, 御前, ょん, お迎え, 好き, ふふ, 部屋, kk, お部屋, 大丈夫, オッ..				
00.18	Kpop - "PRODUCE 101"	78,312	864,011	3.047	495
'produce101jp', 'official_jo1', 'c81125', 'mysta_official', '___komaru', 'Gegg_yna', 'resort_lover', 'zet..				
Produce, japan, Pudeyu, debut, halo, Trainee, love, izone, vote, Osawa, Ando, South Korea, Takumi Kawa..				
produce, japan, プデュ, デビュー, halo, 練習生, 好き, izone, 投票, 大澤, 安藤, 韓国, 川西拓実..				
00.99	Kpop - Others	245,887	2,545,291	3.458	774
'nhk_radiru', 'jp_offclastro', 'MTV_JAPAN', 'bornfreeonekiss', 'offclASTRO', 'CUBE_PTG', 'cubeunited', '..				
Jaejoong, Yoochun, astro, np, jun, future prospective view ii, slowdance, lovecovers, sf9, sweetest, lav..				
ジェジュン, ユチョン, astro, np, jun, 未来予想図 ii, slowdance, lovecovers, sf9, swe..				
01.00	News/hobby - gossip news	1,289,485	13,156,959	2.263	208
'livedoornews', 'kyono_iyashi', 'minnano_dougaww', 'tyomateee', 'yeskiri', 'HEISEI_love_bot', 'gekikawa_..				
Love, Forum, the day before ratio, peing, videos, cartoons, Japan, work, children, www, gifts, photo, wo..				

好き, 質問箱, 前日比, peing, 動画, 漫画, 日本, 仕事, 子供, www, プレゼント, 写真, 女性, 人間, 感じ, ..					
01.01	News/hobby - general news	754,395	21,136,775	1.722	1,510
'YahooNewsTopics', 'nhk_news', 'itm_nlab', 'nhk_seikatsu', 'rei10830349', 'tarareba722', 'gerogeroR', 'm..					
Japan, problem, man, woman, work, children, company, teacher, need, news, cartoons, work, feel, nhk, lov..					
日本, 問題, 人間, 女性, 仕事, 子供, 会社, 先生, 必要, ニュース, 漫画, 作品, 感じ, nhk, 好き, 記事, アニ..					
01.02	News/hobby - Anime	727,834	19,530,657	2.460	730
'fashionpressnet', 'comic_natalie', 'famitsu', 'tkrb_ht', 'appmediafgo', 'shuumai', 'bkub_comic', 'TOUKE..					
Swords boisterous dance, fgo, love, Shinshinsha, cartoon, event, the main enclosure, swords man worker, ..					
刀剣乱舞, fgo, 好き, 審神者, 漫画, イベント, 本丸, 刀剣男士, 先生, キャラ, 映画, オタク, とうらぶ, 感じ, 公..					
01.03	News/hobby - Anime(children)	344,928	3,858,587	2.354	734
'MomentsJapan', 'takaratomytoys', 'Story_terrorV2', 'mtmtSF', 'shoko55mmts', 'oscarnoyukue', 'TMR15', 's..					
Movie, Kamen Rider, Kamen Rider rehmannia, love, work, public, rider, nitiasa, special effects, director..					
映画, 仮面ライダー, 仮面ライダージオウ, 好き, 作品, 公開, ライダー, nitiasa, 特撮, 監督, 登場, ジオウ, 発売..					
01.04	News/hobby - Handcraft	195,256	1,251,351	3.010	182
'k_r_r_l_l_', 'hiroshisj', 'vanity_temple', 'aws_official', 'UenoZooGardens', 'hanamomoact', 'shiitake79..					
Work, exhibition, photo, love, hold, lovely, sale, kimono, illustration, doll, felt, writer, please, par..					
作品, 展示, 写真, 好き, 開催, 素敵, 販売, 着物, イラスト, ドール, 感じ, 作家, お願い, 参加, 出展, イベント,..					
01.05	News/hobby - Train	121,444	1,843,951	2.604	903
'UN_NERV', 'wni_jp', 'earthquake_jp', 'hankyu_ex', 'tetsudoshimbun', 'Kashiken_N', 'Trainfo_', 'plarail_..					
Organization, the day before ratio, vehicle, train, railway, express, photographing, operation, operatio..					
編成, 前日比, 車両, 列車, 鉄道, 特急, 撮影, 運用, 運転, 乗車, 電車, 試運転, 回送, at, in, 急行, 写真,..					
01.06	News/hobby - Anime("JoJo")	103,891	1,272,822	2.510	649
'anime_jojo', 'kidocch1', 'kamuy_anime', 'kamuy_official', 'medicos_et_j', 'askavoltaire1', 'jojopp_offi..					
Jojo, Bucharati, love, Ogata, Giorno, jojo, UketamawaTaro, cartoon, teacher, dio, Golden Kamui, risotto,..					
ジョジョ, ブチャラティ, 好き, 尾形, ジョルノ, jojo, 承太郎, 漫画, 先生, dio, ゴールデンカムイ, リゾット, ギ..					
01.07	News/hobby - Ilustration	68,209	675,790	4.674	825
'cononoc', 'COMITIAofficial', 'shiganai91', 'Tenpure_Nekoka', 'sosaktagtagtag', 'kakka_ekaki', 'mizukita..					
Love, creativity, character, cs, feeling, planning, out, exchange, teacher, man, relationship, cartoon, ..					
好き, 創作, キャラ, cs, 感じ, 企画, うち, 交流, 先生, 人間, 関係, 漫画, picrew, 大丈夫, 作品, 名前,..					
01.08	News/hobby - Boardgame	63,389	733,760	3.582	875
'realdgame', 'perry_trpg', 'kaityouhimegami', 'inouekari', 'K_TRPGonly', 'namakoz01', 'trpgstudio', 'T_M..					
Scenario, trpg, pl, board games, kp, coc, games, search, love, pc, Bodoge, character, gm, feeling, ho, p..					
シナリオ, trpg, pl, ボードゲーム, kp, coc, ゲーム, 探索, 好き, pc, ボドゲ, キャラ, gm, 感じ, h..					
01.09	News/hobby - "Disney"	62,659	547,024	2.801	1,185
'TDR_PR', 'disneyjp', 'DtimesJP', 'MezzoMikiD', 'DtimesDelicious', 'disneychanneljp', 'Pretty_hina_', 'D..					
Tdr, Mickey, Disney, Minnie, fancy dress, Park, Gris, like, minions, now, Goofy, show, Tokyo Disney Sea,..					
tdr, ミッキー, ディズニー, ミニー, 仮装, パーク, グリ, 好き, 手下, now, グーフィー, ショー, 東京ディズニーシ..					
01.10	News/hobby - Anime("Konan")	55,571	695,254	2.346	838
'kinro_ntv', 'kyoani', 'ENma_Dororon', 'torushome', 'conan_file', 'lupin_anime', 'daken_r', 'zerotea_fil..					
Detective Conan, Conan, Akai, Amuro, Furuya, love, Kid, oxalic, Prussian blue, sewing, teacher, Kid, new..					
名探偵コナン, コナン, 赤井, 安室, 降谷, 好き, キッド, シュウ, 紺青, ぬい, 先生, 怪盗キッド, 新刊, 漫画, 原稿,..					
01.11	News/hobby - Nursing	53,657	247,863	4.521	1,161
'kaochan64252585', 'whitephage1515', 'zurukan2018', 'TFE1207', 'chou_kaorinn', 'm181610', 'Tactini', '_L..					
Forum, peing, compared with the day before, love, work, nurse, human, university, feeling, boyfriend, li..					
質問箱, peing, 前日比, 好き, 仕事, 看護師, 人間, 大学, 感じ, 彼氏, 人生, 質問, 勉強, 気持ち, 結婚, 問題..					
01.99	News/hobby - Others	213,955	1,815,419	3.273	693
'hirasawa', 'DQ_PR', 'trms_umee', 'FFXVJP', 'zakutohachigaun', '34thm19', 'karetakoe', 'mupyyyyy', 'SCPP..					
Kemurikusa, Sabage, love, feel, work, game, photo, teacher, mackerel, son, participation, characters, eq..					
ケムリクサ, サバゲー, 好き, 感じ, 仕事, ゲーム, 写真, 先生, サバ, 息子, 参加, キャラ, 装備, うち, イベント, ..					
02.00	Game(male) - "Smash Brothers"	552,357	3,819,915	2.608	250
'purinharumaki', 'Nintendo', 'Pokemon_cojp', 'moa151', 'p_kouhou', 'SmashBrosJP', 'hanari0716', 'randoms..					
Smash Bros., Smash Bros. sp, pokemon, nintendo, character, switch, the, Kirby, Fuka Yukitsuki, like, fe,..					
スマブラ, スマブラ sp, ポケモン, nintendo, キャラ, switch, the, カービィ, 風花雪月, 好き, fe, イ..					
02.01	Game(male) - "Love Live"	527,603	7,253,493	2.095	673

’bang_dream_gbp’, ’bang_dream_info’, ’LLAS_STAFF’, ’starlightrelive’, ’atsushilonboo’, ’LoveLive_staff’,..					
Love Live, Bandori, aqours, campaign, Galle, lovelive, applicants, gifts, Sukusuta, Christmas box campai..					
ラブライブ, バンドリ, aqours, キャンペーン, ガル, lovelive, 応募, プレゼント, スクスタ, クリスマスボックス..					
02.02	Game(male) - Shooting game with Girl	486,635	15,525,631	1.530	1,425
’Ixy’, ’mashiron1020’, ’pageratta’, ’KSUWABE’, ’syatey_12’, ’udon0531’, ’Fate_SN_Anime’, ’lalalalack’, ’..					
Fgo, Azul lane, swimsuit, fatego, new book, cartoon, illustrations, tits, ship it, Dorufuro, girl, fate,..					
fgo, アズールレーン, 水着, fatego, 新刊, 漫画, イラスト, おっぱい, 艦これ, ドルフロ, 少女, fate, アズ..					
02.03	Game(male) - Rhythm game with girl	318,427	4,122,966	2.416	897
’33kitta’, ’nenesamagatizei’, ’hukkatunoyuyuta’, ’kinkyunoyuyuta’, ’tapiokan0623’, ’negi_haruba’, ’pad_s..					
Bandori, the day before ratio, anime, gal, five equal parts of the bride, everyone, birth Festival, Foru..					
バンドリ, 前日比, アニメ, ガル, 五等分の花嫁, 全員, 生誕祭, 質問箱, 履歴書, 誕生日, peing, ドリーマー, プレゼ..					
02.04	Game(male) - Vtuber	253,895	6,427,054	2.257	2,494
’nijisanji_app’, ’g9v9g_mirei’, ’towakisekiv’, ’MitoTsukino’, ’shirakamifubuki’, ’OtogibaraEra’, ’D_W_Un..					
Vtuber, delivery, rainbow disaster, video, youtube, virtual, live, Hororaibu, collaboration, game, chann..					
vtuber, 配信, にじさんじ, 動画, youtube, バーチャル, 生放送, ホロライブ, コラボ, ゲーム, チャンネル, 好..					
02.05	Game(male) - Card game	239,883	2,781,607	2.725	414
’shadowverse_jp’, ’YuGiOh_OCG_INFO’, ’corocoro_tw’, ’Tiziano_Craft’, ’mokouliszt’, ’niconicotanktop’, ’h..					
Deck, cards, Yu-Gi-Oh, Pokemon, Pokeka, Shadoba, Forum, gifts, campaign, shadow Bath, purchase, construc..					
デッキ, カード, 遊戯王, ポケモン, ポケカ, シャドバ, 質問箱, プレゼント, キャンペーン, シャドウバース, 買取, 構築, ..					
02.06	Game(male) - Simulation Game ”The Idolmaster”	218,842	6,871,624	2.000	937
’imascg_stage’, ’imasml_theater’, ’imassc_official’, ’imas_official’, ’imas_DB’, ’arimewasshoi’, ’blue_y..					
Mirishita, Deresute, idle, Shanimasu, producer, birth Festival, Idol Master, Deremasu, Imus, general ele..					
ミリシタ, デレステ, アイドル, シャニマス, プロデューサー, 生誕祭, アイドルマスター, デレマス, アイマス, 総選挙, ssr..					
02.07	Game(male) - music game	190,945	2,362,358	2.929	804
’chunithm’, ’ongeki_official’, ’Falcon_chnpa’, ’cametek’, ’Eromanga_atume’, ’CODAKUMI’, ’masakinman7322’..					
SCORE, the day before ratio, sound game, Volte, Ongeki, Forum, ZenRyo, dj, peing, arcade, Nobu, love, ga..					
譜面, 前日比, 音ゲー, ボルテ, オンゲキ, 質問箱, 全良, dj, peing, ゲーセン, のぶ, 好き, ゲーム, 楽曲, 感..					
02.08	Game(male) - ”Granblue Fantasy”	135,523	1,482,960	2.163	1,225
’granbluefantasy’, ’Granblue_GW’, ’guran_mimumemo’, ’dasazyake’, ’hiyopi’, ’nupnosenshi’, ’kusokaisetu_g..					
Recruitment, lv, id, war, Guraburu, Battlefield, rpg, Magna, smartphone, character, proto Bahamut, Grand..					
参加者募集, lv, id, 参戦, グラブル, 古戦場, rpg, マグナ, スマホ, キャラ, プロトバハムート, グランブルーファン..					
02.09	Game(male) - ”Gundam”	132,844	1,883,109	2.995	931
’MH_official_JP’, ’p8HMIUHYW1KUF6c’, ’CALAMI_DESTINY’, ’figsoku’, ’redol_0H0w0M0’, ’Chris_novas’, ’zabu7..					
Gear, Alice, Gundam, aircraft, characters, ps4, Batoope, games, Ekuba, girl, weapon, Gundam, fgo, Forum,..					
ギア, アリス, ガンダム, 機体, キャラ, ps4, バトオペ, ゲーム, エクバ, ガール, 武器, ガンプラ, fgo, 質問箱,..					
02.10	Game(male) - ”Kemono Friends”	128,284	2,107,990	2.146	923
’onj334ngo’, ’Vanila_Ice_Ice’, ’irodori7’, ’jidan_no_jouken’, ’S___Frandle’, ’ayn398’, ’honey_burst’, ’Fo..					
Midnight seriously painting 60 minutes Ipponshobu, east, beast Friends, the day before ratio, east proje..					
深夜の真剣お絵描き 60 分一本勝負, 東方, けものフレンズ, 前日比, 東方 project, 魔理沙, けもフレ, 霊夢, 質問箱, 例大..					
02.11	Game(male) - Smartphone Game(”Compus”)	111,427	1,597,366	2.471	1,073
’cps_niconico’, ’Matsu_Kusarine’, ’famitsuApp’, ’nanawoakari’, ’TYOMHHN’, ’SuamaUMUM3’, ’baru_san12345’,..					
Compass, Forum, peing, fixed, resume, Thorn, Adam, painting, Lyrica, question, season, Marcos, love, all..					
コンパス, 質問箱, peing, 固定, 履歴書, ソーン, アダム, お絵描き, リリカ, 質問, シーズン, マルコス, 好き, 味..					
02.12	Game(male) - ”tenka_zan”	93,467	1,030,792	3.245	723
’priconne_redive’, ’key_official’, ’yuzusoft’, ’flower_staff’, ’qruppo’, ’tenka_zan’, ’sprite_fairys’, ’..					
Purikone, Tenhana hundred sword, characters, campaign, lv, gifts, knight, compared with the day before, ..					
プリコネ, 天華百剣, キャラ, キャンペーン, lv, プレゼント, 騎士, 前日比, id, ガチャ, 参加者募集, クラバト, ゲー..					
02.13	Game(male) - Battleship game with girl	90,147	1,566,076	2.332	1,176
’azurlane_staff’, ’KanColle_STAFF’, ’pakkopako’, ’idonum’, ’tamayan22’, ’C2_STAFF’, ’docuwo’, ’hellmayug..					
Ship this, painting 60 minutes Ipponshobu, Admiral, seriously, azure lane, Garupan, Kanmusume, wwyxrgnna..					
艦これ, お絵描き 60 分一本勝負, 提督, 真剣, アズールレーン, ガルパン, 艦娘, wwyxrgnnaf, 作戦, 司令官, 時雨,..					
02.14	Game(male) - ”PSO2”	61,674	970,888	2.837	2,097
’sega_pso2’, ’sega_idola’, ’tencho_tsurusan’, ’project_ge’, ’zyagaente01’, ’RUIS_MAX’, ’PSPTAKEO’, ’Ryun..					
Pso2, ARCS, put a ss because maintenance of the day, ss, maintenance, Forum, characters, weapons, peing,..					

pso2, アークス, メンテの日なので ss を貼る, ss, メンテ, 質問箱, キャラ, 武器, peing, フォロワー, ps4, ..					
02.15	Game(male) - "SOULCALIBUR"	53,556	547,042	2.793	1,401
'gamenohanashi', 'ARCSY_Event', 'heartia2', 'Harada_TEKKEN', '4eajt', 'GUILTYGEAR_PR', 'BLAZBLUE_PR', 'R..					
Match-up, character, ps4, fighting game, share, combo, game, tournament, delivery, Soul Calibur, guard, ..					
対戦, キャラ, ps4, 格ゲー, share, コンボ, ゲーム, 大会, 配信, ソウルキャリバー, ガード, スト 5, ggxrd..					
02.16	Game(male) - "Hatsune Miku"	53,006	928,319	4.483	1,545
'ShirayukiTowa', 'cfm_miku', 'take_samurai1', 'niijimaakiichi', 'pjd_sega', 'kakuriyon', 'magicalmirai',..					
Phosphorus, Hatsune Miku, Miku, Yukari, sm, Akari, alone with, Kinha, Yuitsuki Yukari, snow Miku, voicer..					
リン, 初音ミク, ミク, ゆかり, sm, あかり, きり, 琴葉, 結月ゆかり, 雪ミク, voiceroid, ドール, マジカルミ..					
02.99	Game(male) - Others	65,541	629,208	3.786	1,779
'fgoproject', 'hitodepyonpyon', 'Mashumaro_114', 'Kaycfgo', 'mahouotome_info', 'syaorin_2009', 'sgs_pr',..					
Fgo, Magireko, friendship, fate, events, dress, Chaldean, Forum, character, circling, like, Gacha, magic..					
fgo, マギレコ, フレンド, fate, イベント, 礼装, カルデア, 質問箱, キャラ, 周回, 好き, ガチャ, 魔法少女, ま..					
03.00	Campaign - 0	1,236,728	6,408,468	3.287	146
'akiko_lawson', 'NitoriOfficial', 'McDonaldsJapan', 'Kirin_Company', 'AEON_JAPAN', 'GalaxyMobileJP', 'JA..					
Campaign, coupon, applicants, lottery, challenge, participation, gifts, 20 yen, losing, opportunity, pos..					
キャンペーン, クーポン, 応募, 抽選, チャレンジ, 参加, プレゼント, 20 円, ハズレ, チャンス, 投稿, はずれ, リツイー..					
03.01	Campaign - 1	428,723	1,074,145	2.062	81
'misakism13', 'xxsmsmsmxx', 'guccl_men_men', 'POINTJP_TSUTOMU', 'asukakiraran', 'TheVambi', 'ya_mi_ya__m..					
Gifts planning, gifts, how to apply, winning, dm, retweets, 1 million yen, cash, lottery, applicants, se..					
プレゼント企画, プレゼント, 応募方法, 当選, dm, リツイート, 100 万円, 現金, 抽選, 応募, 販売, amp, 当選者,..					
03.02	Campaign - 2	409,816	604,415	1.780	103
'poke_times', 'PlayStation_jp', '7taizai_GrandX', 'monst_campaign', 'GirlsFrontline', 'Touhou_LW', 'dokk..					
Campaign, gifts, lottery, Memorial, applicants, winning, being held, Dokkan battle, scratches, participa..					
キャンペーン, プレゼント, 抽選, 記念, 応募, 当選, 開催中, ドッカンバトル, キズ, 参加, サイン色紙, 開催, 動画, 応..					
03.03	Campaign - 3	312,788	328,415	4.441	51
'yousuck2020', 'zozojp', 'ChocoplaOsada', 'yousuck2019', 'monomart_style', 'genki_ochan', 'mzsupercar', ..					
Gifts, campaign, lottery, retweets, applicants, participants recruited, id, lv, How to Apply, Dokkan bat..					
プレゼント, キャンペーン, 抽選, リツイート, 応募, 参加者募集, id, lv, 応募方法, ドッカンバトル, 参戦, 配信中, ..					
03.04	Campaign - 4	267,179	7,027,324	1.575	139
'ExciteJapan', 'KADOKAWA_denshi', 'DAMch_Official', 'daikinstreamer', 'Seiyu_Japan', 'donki_donki', 'wir..					
Campaign, gifts, lottery, application, application method, posts, retweets, amp, amazon gift certificate..					
キャンペーン, プレゼント, 抽選, 応募, 応募方法, 投稿, リツイート, amp, amazon ギフト券, 当選, 参加, チャンス..					
03.05	Campaign - 5	176,554	254,566	2.063	868
'dominos_JP', 'pepsi_jpn', 'dospara_web', 'PRORS3', 'wacom_info_jp', 'ASUSROGJP', 'pommier1484', 'Monste..					
Participant recruitment, gifts, lv, war, id, campaign, contest, lottery, Keisuke Honda, applicants, retw..					
参加者募集, プレゼント, lv, 参戦, id, キャンペーン, 勝負, 抽選, 本田圭佑, 応募, リツイート, 残念, 勝利, am..					
03.06	Campaign - 6	142,145	332,154	2.581	101
'sinoalice_jp', 'nico_nico_news', 'nittele_da_bear', 'nicoanime_PR', 'PUBGMOBILE_JP', 'iijmio', 'Disgaea..					
Campaign, lottery, gifts, amazon gift certificates, post, How to Apply, apply, Memorial, retweets, amp, ..					
キャンペーン, 抽選, プレゼント, amazon ギフト券, 投稿, 応募方法, 応募, 記念, リツイート, amp, 参加方法, ギフ..					
03.07	Campaign - 7	111,722	142,752	3.988	198
'QUICPay_PR', 'Starbucks_J', 'AVG_JP', 'disneygames_jp', 'disneystudiojp', 'SpidermanfilmJP', 'starwarsj..					
Gifts, campaign, applicants, in a few days, lottery, retweets, participation, winning, post, dm, Memoria..					
プレゼント, キャンペーン, 応募, 近日中, 抽選, リツイート, 参加, 当選, 投稿, dm, 記念, お送り, スパイダーマン, ..					
03.08	Campaign - 8	108,091	200,835	2.118	133
'mayla_classic', 'SHIBUYA109NET', 'LocoLoco0011', 'opera_cosmetics', 'voguejp', 'NieR_tokyo', 'kyo_miori..					
Gifts, application, campaign, amp, 365 days, completion, mayla, classic, item, how to apply, present pla..					
プレゼント, 応募, キャンペーン, amp, 365 日, 完了, mayla, classic, アイテム, 応募方法, プレゼント企画..					
03.09	Campaign - 9	80,623	655,796	4.034	737
'teddystaff', 'nine_d2015', 'puronmy', 'clovisbaby', 'BABIESRUS_JP', 'mamaaka0102', 'nac_ikuji', 'ryo_ay..					
Husband, son, baby, baby food, breast-feeding, hug, pregnancy, out, stomach, Mom, birth, children, milk,..					
旦那, 息子, 赤ちゃん, 離乳食, 授乳, 抱っこ, 妊娠, うち, お腹, ママ, 出産, 子供, ミルク, 育児, 旦那さん, 母乳..					
03.99	Campaign - others	299,746	785,086	2.617	454

’mollyfantasy_of’, ’Qoo_Japan’, ’rilakkuma_gyr’, ’LINEMOBILE_JP’, ’kaori_saison’, ’tvasahi_cs’, ’aquiz_s..					
Gifts, campaign, lottery, retweets, applicants, faucet, how to apply, amp, quail, post, free, karaoke Ma..					
プレゼント, キャンペーン, 抽選, リツイート, 応募, 蛇口, 応募方法, amp, うずら, 投稿, 無料, カラオケマック, 仮想..					
04.00	Teenagers - Youtuber 1	247,720	1,512,530	2.194	792
’hajimesyacho’, ’hikakin’, ’kantamizutamari’, ’RytoSle2’, ’miztamari_nikki’, ’uuum_news’, ’soraciavntis’..					
Video, Kanta, water reservoir bond, Fishers, Abanti’s, today’s video, youtube, Tokai on-air, youtuber, T..					
動画, カンタ, 水溜りボンド, フィッシャーズ, アバンティーズ, 今日の動画, youtube, 東海オンエア, youtuber, ..					
04.01	Teenagers - Rock bands 1	235,581	1,917,057	2.582	410
’Yahoo_GYAO’, ’YojiNoda1’, ’okazaki_taiiku’, ’skream_japan’, ’pazu_official’, ’yabaT_koyacial’, ’tenkino..					
Rock, band, tour, ticket, rock, love, EmiEmi, Forum, sumika, radwimps, peing, fm802, best, favorite pers..					
ロック, バンド, ツアー, チケット, rock, 好き, 笑笑, 質問箱, sumika, radwimps, peing, fm80..					
04.02	Teenagers - Teenage girl fashion	220,213	866,261	2.173	869
’Popteen_cgw’, ’udon_lov’, ’radran10’, ’HeiseiiSaigoo’, ’PRINZ_Leipzig’, ’neo_neo66’, ’Mk0104Sig’, ’Popt..					
Popu, Neo, popteen cover girl war, war, vote, Forum, retweets, Reita, Yuna, questions, popteen, peing, p..					
ポプ, ねお, popteen カバーガール戦争, 戦争, 投票, 質問箱, リツイート, れいた, ゆな, 質問, popteen, pe..					
04.03	Teenagers - Rock bands 2	127,021	767,707	2.493	500
’uchikubigokumon’, ’mwamjapan’, ’MTH_OFFICIAL’, ’gekirock’, ’Pinakano’, ’summer_sonic’, ’a_yanaka’, ’ken..					
Band, ticket, the, tour, tour, best, performers, dj, love, fun, work, announcement, Osaka, live, Fes, pl..					
バンド, チケット, the, tour, ツアー, 最高, 出演, dj, 好き, 楽しみ, 仕事, 発表, 大阪, live, フェス..					
04.04	Teenagers - Youtuber 2	123,147	830,677	2.314	644
’sankoiti_offici’, ’My_kwk_N’, ’yorihito_vine’, ’JINJIN1027’, ’teokun711’, ’skypeace_teojin’, ’JINSJINS1..					
Sky piece, Theo, Jintan, Miyagawa-kun, descriptor, Miya, videos, EmiEmi, questions, Foroba, Forum, fun, ..					
スカイピース, テオ, じんたん, みやかわくん, リプ, みや, 動画, 笑笑, 質問, フォロバ, 質問箱, 楽しみ, プレゼント, ..					
04.05	Teenagers - Indies bands	122,467	504,882	3.203	1,223
’ichika_mo’, ’ui_i_i_’, ’sigure_official’, ’____hizumi____’, ’some0658’, ’pinkhage2’, ’famifamimario’, ’to..					
Band, Forum, peing, guitar, bass, the, cast, mv, Shimokitazawa, like, questions, best, ban, open, music,..					
バンド, 質問箱, peing, ギター, ベース, the, 出演, mv, 下北沢, 好き, 質問, 最高, 解禁, open, 音楽..					
04.06	Teenagers - University admission	122,052	381,565	2.908	1,226
’sutehageyoutube’, ’nikuinem’, ’BreakthroughSSK’, ’CenterRemaining’, ’superschoolsbot’, ’sute_kame’, ’su..					
Study, Forum, peing, mathematics, University, studyplus, questions, English, lessons, exam, pass, Waseda..					
勉強, 質問箱, peing, 数学, 大学, studyplus, 質問, 英語, 授業, 受験, 合格, 早稲田, 問題, 慶應, テ..					
04.07	Teenagers - ”Osaka”	115,363	283,215	3.535	1,533
’scmvbss_hahaha’, ’mvMHAhhX7nTAHsv’, ’ishibassi519i’, ’poco_crescendo’, ’happa_eight’, ’caffeine142mg’, ..					
Forum, peing, questions, bytes, Honma, anonymous, in recruiting, university, teaching, llll, love, EmiEm..					
質問箱, peing, 質問, バイト, ほんま, 匿名, 募集中, 大学, 授業, llll, 好き, 笑笑, バンド, プレゼント, ..					
04.08	Teenagers - ”Kyusyu”	105,030	282,266	4.801	956
’mashashico’, ’leon_kit1560’, ’yuji_qualia0’, ’queblick’, ’george91617’, ’_kubotakai_’, ’tops_inc’, ’___k..					
Forum, peing, questions, band, Fukuoka, bytes, anonymous, in recruiting, love, EmiEmi, Buuuuu, Seriously..					
質問箱, peing, 質問, バンド, 福岡, バイト, 匿名, 募集中, 好き, 笑笑, ﾌﾞｳｳｳｳ, まじ, プレゼント, 単位,..					
04.09	Teenagers - ”Tohoku”	77,431	198,988	4.087	724
’explode_high’, ’CLUB_RIVERST’, ’masayasu_zan’, ’outline1’, ’happymylife0712’, ’taku_1826’, ’MagNetHIROS..					
Forum, peing, questions, fresh, box, anonymous, in recruiting, love, bytes, band, answer, EmiEmi, Seriou..					
質問箱, peing, 質問, fresh, box, 匿名, 募集中, 好き, バイト, バンド, 回答, 笑笑, まじ, プレゼント,..					
04.10	Teenagers - ”Nagoya”	77,418	223,003	3.052	1,708
’Maji_Oisy_Omiz’, ’NU_yakimikan’, ’cookie_IWT’, ’ZNTGNHTS’, ’itoharu_666’, ’hal_nagoyauni’, ’TsushimaKit..					
Forum, peing, fresh, question, box, anonymous, in recruiting, compared with the day before, bytes, like,..					
質問箱, peing, fresh, 質問, box, 匿名, 募集中, 前日比, バイト, 好き, 回答, 大学, 笑笑, 先輩, 高校..					
04.11	Teenagers - Youtuber 3	61,350	315,208	2.188	1,947
’DJ_Shacho’, ’karuma3960’, ’in_FoyLand’, ’DJ_Shacho2’, ’kinnpatuhikaru’, ’represen_chikyu’, ’Kyoooheiz’,..					
Rep, Hikaru, Foy, Earth, Forum, GinFutoshi, videos, questions, peing, president, EmiEmi, love, gifts, Fo..					
レペゼン, ヒカル, ふぉい, 地球, 質問箱, 銀太, 動画, 質問, peing, 社長, 笑笑, 好き, プレゼント, フォロバ, ..					
04.12	Teenagers - ”Hokkaido”	60,683	156,280	4.274	718
’kyremon’, ’uranai_yagizaa’, ’dosanko_loveboy’, ’asariFPS’, ’mochi_future’, ’ba_ra_che_tech’, ’norio3663..					
Forum, peing, fresh, question, box, EmiEmi, anonymous, in recruiting, Sapporo, bytes, Hokkaido Universit..					

<table>
<tr><td colspan="5">質問箱, peing, fresh, 質問, box, 笑笑, 匿名, 募集中, 札幌, バイト, 北大, 回答, 好き, プレゼント, ..</td></tr>
<tr><td>04.13</td><td>Teenagers - ”Osaka-2”</td><td>60,583</td><td>143,073</td><td>4.505</td><td>430</td></tr>
<tr><td colspan="6">’kuroshio_287’, ’kawuo01’, ’gotoriiiita’, ’y_mame_y’, ’tamate_6’, ’pepppppushi’, ’N___Summer___’, ’MAHOTO_..</td></tr>
<tr><td colspan="6">Forum, peing, questions, fresh, box, anonymous, in recruiting, laughter, EmiEmi, Honma, answer, love, gi..</td></tr>
<tr><td colspan="6">質問箱, peing, 質問, fresh, box, 匿名, 募集中, 爆笑, 笑笑, ほんま, 回答, 好き, プレゼント, 高校, ..</td></tr>
<tr><td>04.14</td><td>Teenagers - Quize</td><td>59,101</td><td>198,649</td><td>2.505</td><td>1,265</td></tr>
<tr><td colspan="6">’bozu_108’, ’syaaaan_’, ’tax_i_’, ’sou_mizukami’, ’QuizKnock’, ’Sugai_Shunki’, ’Kawakami_q’, ’Miracle_Fu..</td></tr>
<tr><td colspan="6">Quizknock, University of Tokyo King, quiz, early push quiz, Forum, Izawa, peing, the correct answer, qui..</td></tr>
<tr><td colspan="6">quizknock, 東大王, クイズ, 早押しクイズ, 質問箱, 伊沢, peing, 正解, quiz, 質問, はや, 動画, 問題..</td></tr>
<tr><td>04.15</td><td>Teenagers - Music</td><td>57,726</td><td>392,668</td><td>2.795</td><td>1,588</td></tr>
<tr><td colspan="6">’hachi_08’, ’rockinon_com’, ’MatsuiRyosuke’, ’boc_chama’, ’hitorieJP’, ’reissuerecords’, ’boc_official_’..</td></tr>
<tr><td colspan="6">Yonezu, KENSHI YONEZU, bump, ghost, love, horses and deer, auroraark, aurora, lemon, Fuji-kun, fun, nice..</td></tr>
<tr><td colspan="6">米津, 米津玄師, bump, 幽霊, 好き, 馬と鹿, auroraark, aurora, lemon, 藤くん, 楽しみ, 素敵, ..</td></tr>
<tr><td>04.16</td><td>Teenagers - ”Chubu”</td><td>51,438</td><td>107,449</td><td>4.421</td><td>668</td></tr>
<tr><td colspan="6">’Am_drxx09’, ’Hashida_____’, ’my8yeh’, ’C28H24N2O7_knct’, ’yagidehito’, ’boc_snoopy182’, ’ramengonzo’, ’to..</td></tr>
<tr><td colspan="6">Forum, peing, question, anonymous, in recruiting, bytes, EmiEmi, fresh, box, love, Okayama, gifts, answe..</td></tr>
<tr><td colspan="6">質問箱, peing, 質問, 匿名, 募集中, バイト, 笑笑, fresh, box, 好き, 岡山, プレゼント, 回答, まじ, ..</td></tr>
<tr><td>04.99</td><td>Teenagers - others</td><td>886,157</td><td>3,540,681</td><td>2.910</td><td>177</td></tr>
<tr><td colspan="6">’Satoru_191’, ’aimyonGtter’, ’zutomayo’, ’saori_skow’, ’KingGnu_JP’, ’UVERworld_dR2’, ’sugales_noah’, ’S..</td></tr>
<tr><td colspan="6">Forum, peing, questions, like, EmiEmi, compared with the day before, anonymous, perfume, best, gifts, re..</td></tr>
<tr><td colspan="6">質問箱, peing, 質問, 好き, 笑笑, 前日比, 匿名, perfume, 最高, プレゼント, 募集中, リトグリ, 楽しみ, ..</td></tr>
<tr><td>05.00</td><td>Hobby/etc - Job recruiting</td><td>273,522</td><td>2,758,344</td><td>2.326</td><td>3,835</td></tr>
<tr><td colspan="6">’manabubannai’, ’minowanowa’, ’mutekinokasan’, ’PositiveSalon’, ’kumapadsense’, ’ha_chu’, ’zubora_tsuma’..</td></tr>
<tr><td colspan="6">Mutual follower, followers, accounts, mutual, fixed, Foroba, everyone, blog, sougofollow, support, follo..</td></tr>
<tr><td colspan="6">相互フォロー, フォロワー, アカウント, 相互, 固定, フォロバ, 全員, ブログ, sougofollow, 支援, followm..</td></tr>
<tr><td>05.01</td><td>Hobby/etc - Photograph</td><td>262,860</td><td>2,130,032</td><td>3.290</td><td>1,451</td></tr>
<tr><td colspan="6">’aveirjapan’, ’reiwa_chann’, ’joker_masiro’, ’tutiyak’, ’utakoww’, ’tenkunofuji’, ’hacci_0731’, ’MiiiMii..</td></tr>
<tr><td colspan="6">Photo, finder, world, favorite person, nice, Imasora, line stamp, please, stay, lunch, gentle, the, opti..</td></tr>
<tr><td colspan="6">写真, ファインダー, 世界, 好きな人, 素敵, イマソラ, line スタンプ, お願い, お過ごし, 弁当, 穏やか, the, 楽天..</td></tr>
<tr><td>05.02</td><td>Hobby/etc - Streaming</td><td>220,941</td><td>3,264,084</td><td>2.432</td><td>533</td></tr>
<tr><td colspan="6">’nijimasu_rinrin’, ’popnrolltv’, ’knzk803’, ’TIP_TIF_staff’, ’misaki_natsume_’, ’yuina_2780’, ’tikaidolm..</td></tr>
<tr><td colspan="6">During the delivery, idle, cheki, performers, booking, distribution, product sales, events, support, Sur..</td></tr>
<tr><td colspan="6">配信中, アイドル, チェキ, 出演, 予約, 配信, 物販, イベント, 応援, スリジエ, 楽しみ, live, 渋谷, showro..</td></tr>
<tr><td>05.03</td><td>Hobby/etc - Night Job recruiting</td><td>218,006</td><td>1,325,822</td><td>2.077</td><td>759</td></tr>
<tr><td colspan="6">’pamyurin’, ’EIMI_FUKADA’, ’kawasaki___aya’, ’elaiza_ikd’, ’yukakuramoti’, ’yua_mikami’, ’ai_uehara_ex’, ..</td></tr>
<tr><td colspan="6">Men Este, customs, attendance, therapist, booking, guide, photo session, Este, Deriheru, jobs, vr, fanza..</td></tr>
<tr><td colspan="6">メンズエステ, 風俗, 出勤, セラピスト, 予約, 案内, 撮影会, エステ, デリヘル, 求人, vr, fanza, イベント, 新..</td></tr>
<tr><td>05.04</td><td>Hobby/etc - ”AKB48”</td><td>185,734</td><td>2,547,487</td><td>2.129</td><td>949</td></tr>
<tr><td colspan="6">’345___chan’, ’SayakaNeon’, ’0220nicole’, ’k3po5gouki’, ’maho_yamaguchi’, ’Yukiriiiin___K’, ’kojiharunyan’..</td></tr>
<tr><td colspan="6">Akb48, ske48, during the delivery, performance, handshake meeting, nmb48, stu48, hkt48, ngt48, graduatio..</td></tr>
<tr><td colspan="6">akb48, ske48, 配信中, 公演, 握手会, nmb48, stu48, hkt48, ngt48, 卒業, メンバー, 山口真..</td></tr>
<tr><td>05.05</td><td>Hobby/etc - Dog/cat</td><td>162,619</td><td>2,168,910</td><td>2.168</td><td>1,891</td></tr>
<tr><td colspan="6">’neko2time’, ’otakunews6398’, ’mofunekoc1’, ’ehara_hiroyuki_’, ’cutest_animal1’, ’iyashiworld’, ’best1_m..</td></tr>
<tr><td colspan="6">Foster parents, lost, protection, diffusion hope, the owner, slaughter, collar, lost dog, recruitment, p..</td></tr>
<tr><td colspan="6">里親, 迷子, 保護, 拡散希望, 飼い主, 殺処分, 首輪, 迷い犬, 募集, 保健所, 期限, お願い, 収容, 動物, ねこ, ペッ..</td></tr>
<tr><td>05.06</td><td>Hobby/etc - Indies bands</td><td>114,350</td><td>769,318</td><td>3.668</td><td>389</td></tr>
<tr><td colspan="6">’getters_iida’, ’time720’, ’berrygoodman88’, ’MOCA_OSAKA’, ’tokuma_japan’, ’826aska_STAGEA’, ’HAZZIE3’, ..</td></tr>
<tr><td colspan="6">Live, performers, one-man, during the delivery, delivery, one-man live, fun, ticket, open, start, reserv..</td></tr>
<tr><td colspan="6">live, 出演, ワンマン, 配信中, 配信, ワンマンライブ, 楽しみ, チケット, open, start, 予約, 音楽, 素敵,..</td></tr>
<tr><td>05.07</td><td>Hobby/etc - ”Bish(Pop star)”</td><td>100,794</td><td>1,108,666</td><td>2.449</td><td>715</td></tr>
<tr><td colspan="6">’BiSHidol’, ’YahooNewsYuru’, ’KatyHanpen’, ’aNo2mass’, ’TimelineNews_tv’, ’aina_BiSH’, ’mogatanpe’, ’Chi..</td></tr>
<tr><td colspan="6">Bish, bis, idle, cheki, empire, love, ticket, one-man, Gyanpare, performers, Ririibe, Shibuya, propagati..</td></tr>
<tr><td colspan="6">bish, bis, アイドル, チェキ, empire, 好き, チケット, ワンマン, ギャンパレ, 出演, リリイベ, 渋谷, でん..</td></tr>
<tr><td>05.08</td><td>Hobby/etc - Professional wrestling</td><td>92,874</td><td>710,738</td><td>2.606</td><td>595</td></tr>
</table>

'njpw1972', 'naoyainoue_410', 'rainmakerXokada', 'njpwworld', 'tanahashi1_100', 'WWEAsuka', 'takerusegaw..				
Athletes, wrestling, tournament, game, njpw, vs, noah, ghc, Korakuen, New Japan Pro Wrestling, Korakuen ..				
選手, プロレス, 大会, 試合, njpw, vs, noah, ghc, 後楽園, 新日本プロレス, 後楽園ホール, rizin, 新..				
05.09	Hobby/etc - Streamer("Ishikawa")	88,324	362,865	3.193	775
'ICCHY8591', 'kppkpp1', 'NORIYUKICAS', 'ChZozozo', 'kk1992kkkk', 'OBtaroooccaas2', 'pococha_jp', 'taroo..				
Delivery, Ishikawa Tengyo, noriradi, during the delivery, dokidoki, Forum, Moi, live, TwitCasting, raw de..				
配信, 石川典行, noriradi, 配信中, dokidoki, 質問箱, モイ, live, ツイキャス, 生配信, peing, ..				
05.10	Hobby/etc - Pachinko	77,748	731,745	2.742	912
'terai_ScooP', 'tetsu0722', 'higenjin', 'usamiiin', 'courage05x2', 'natsumi_scooptv', 'y_datemegane50', ..				
Pachinko, pachislot, slot, Xintai, operation, Uchiko, Sulo, set, jugglers, model, ww, investment, hole, ..				
パチンコ, パチスロ, スロット, 新台, 稼働, 打ち子, スロ, 設定, ジャグラー, 機種, ww, 投資, ホール, 動画, 来店..				
05.11	Hobby/etc - theatre	75,451	560,475	4.455	1,476
'Chisato_Wizard', 'Namio_dao', 'YasakaSaori', 'akiyamayuzuki', 'wonderfulbaseba', 'canary_adachi', 'tsut..				
Performances, rehearsals, during the delivery, stage, reservation, theater, performers, theater, deliver..				
公演, 稽古, 配信中, 舞台, 予約, 劇団, 出演, 観劇, 配信, 演劇, 来場, 作品, 質問箱, 朗読, 皆様, peing, ..				
05.12	Hobby/etc - Streamer(Girl)	73,486	642,815	2.888	1,519
'runa_nkgw', '_st_1972', 'RYUSEI03580565', 'ilkoallexandrof', 'misscd2019_YE01', 'neokamayan', 'yohei_sa..				
'Delivering, Portrait, delivery, photo session, room, model, room, showroom, support, mixchannel, shootin..				
配信中, ポートレート, 配信, 撮影会, ルーム, モデル, room, showroom, 応援, mixchannel, 撮影, ガ..				
05.13	Hobby/etc - Horse racing	61,958	538,020	2.471	701
'netkeiba', 'keibalab', 'Jisou_hou', 'fujitvkeiba', 'gendai_keiba', 'andokatsumi', 'Versailles_Farm', 'u..				
Horse racing, jockey, race, expected, betting, jra, keiba, bicycle race, center, Stakes, stables, two pi..				
競馬, 騎手, レース, 予想, 馬券, jra, keiba, 競輪, 的中, 重賞, 厩舎, 2 着, 出走, 中山, 有馬記念, 大井..				
05.14	Hobby/etc - "Babymetal"	60,182	508,948	2.924	946
'BABYMETAL_JAPAN', 'savage_headbang', 'HMV_Metal', 'saxfather', 'BassistBOH', 'TakayoshiOhmura', 'Downlo..				
Babymetal, metal, metaljapanrecords, Sakura Gakuin, band, cd, guitar, the, sale, information, metal, fac..				
babymetal, metal, metaljapanrecords, さくら学院, バンド, cd, ギター, the, 販売, 情報..				
05.15	Hobby/etc - Ramen	55,656	469,433	4.254	4,458
'Ibaraki_Kouhou', 'rekishichosadan', 'kouhou_mito', '3_syouktaro', 'kansai_noodle', 'susuru_tv', 'amasyo..				
Soup, noodles, soy sauce, noodle, roast pork, buckwheat, anchovy, noodles, anchovy, Chinese noodles, fla..				
スープ, ラーメン, 醤油, つけ麺, チャーシュー, そば, 煮干し, らーめん, 煮干, 中華そば, 旨味, 営業, 限定, 濃厚, ..				
05.16	Hobby/etc - Diet	54,443	283,996	3.371	1,343
'greatsaiyaman02', 'koooarashi', 'Yoshinori_TV', 'sakuratinn316', 'makoto05300530', 'YokokawaNaotaka', '..				
Diet, muscle training, body weight, muscle, effect, training, protein, carbohydrates, calories, training..				
ダイエット, 筋トレ, 体重, 筋肉, 効果, トレーニング, プロテイン, 糖質, カロリー, トレ, 運動, 食事, ジム, 美容, ..				
05.17	Hobby/etc - Pet	51,088	457,645	3.569	971
'modokinatu', 'tonkatsu0209', 'wanivspbao', 'papanikki1', 'piccolocibi', 'healing_rabbit', 'chibita_farm..				
Rabbits, hamsters, pet shops, reptiles, breeding, aquarium, medaka, chinchillas, guinea pigs, cage, out,..				
うさぎ, ハムスター, ペットショップ, 爬虫類, 飼育, 水槽, メダカ, チンチラ, モルモット, ケージ, うち, フェレット, 鈴..				
05.99	Hobby/etc - Others	514,238	4,446,120	2.820	470
'love2chiitan', 'funassyi', 'macegem', '55_kumamon', 'MorningMusumeMg', 'Haruka_Kudo1027', 'heroineSx', ..				
Handmade, minne, peach black, Morning Musume, Angerme, like, update, blog, event, beyooooonds, juice, ph..				
ハンドメイド, minne, ももクロ, モーニング娘, アンジュルム, 好き, 更新, blog, イベント, beyooooonds,..				
06.00	Male Pop stat - "EXILE"	292,153	2,267,862	2.299	507
'modelpress', 'AbemaTV', 'fns_kayousai', 'Mst_com', 'cx_mezamashi', 'ZIP_TV', 'TBSCDTV', 'realsoundjp', ..				
Gene, generations, exile, the third generation, Ryota Katayose, fantastics, the, ldh, Aarashi, Lampe, th..				
gene, generations, exile, 三代目, 片寄涼太, fantastics, the, ldh, 亜嵐, ランペ, t..				
06.01	Male Pop Star - "King and princess"	244,247	2,118,404	2.303	781
'kaguyasama0906', 'Yahoo_JAPAN_PR', 'Johnnysnewinfor', 'uchino_shitsuji', '3_s1c', 'MrKING292523', 'ores..				
Murasaki 耀, Kinpuri, prince, king, plains, Kaito, Ren Nagase, plain Murasaki 耀, Nagase, Takahashi, hihi, a..				
紫耀, キンプリ, prince, king, 平野, 海人, 永瀬廉, 平野紫耀, 永瀬, 髙橋, hihi, amp, 神宮寺勇太, ..				
06.02	Male Pop Star - "SMAP"	238,090	3,267,284	2.009	536
'oricon', 'eiga_natalie', 'cinematoday', 'nikkansports', 'H_KANNA_0203', 'mn_enta', 'mantanweb', 'barks_..				
Shingo Katori, smap, Goro Inagaki, new map, Kusa 彅, Masahiro Nakai, Shingo, Nana, Takuya Kimura, movie, N..				

香取慎吾, smap, 稲垣吾郎, 新しい地図, 草彅, 中居正広, 慎吾, なな, 木村拓哉, 映画, 中居くん, 凪待ち, 中居, b..					
06.03	Male Pop Star - ”SNOWMAN”	226,071	2,760,093	2.216	1,091
’thetvjp’, ’nhk_radirer’, ’islandtv_up’, ’tokyojapan999’, ’YouTubeJapan’, ’J_Jr_Ch’, ’weekly_tvguide’, ’..					
Sixtones, snowman, snow, Naniwa boy, Johnny, travis, nhk Rajira, boys, Jesse, island, Koji, jr, Hokuto, ..					
sixtones, snowman, snow, なにわ男子, ジャニーズ, travis, nhk らじらー, 少年たち, ジェシー, i..					
06.04	Male Pop Star - ”Arashi”	186,257	2,561,843	2.090	883
’arashi5official’, ’Aoki_Genta’, ’junjunmjgirly’, ’nhk_kouhaku’, ’Lunassyi’, ’NetflixJP’, ’ariyoshihiroi..					
Ohno, Aiba, Satoshi Ono, Sho-kun, Sakurai, Jun Matsumoto, Ninomiya, Sho Sakurai, Kazunari Ninomiya, aras..					
大野, 相葉, 大野智, 翔くん, 櫻井, 松本潤, 二宮, 櫻井翔, 二宮和也, arashi, 松本, ニノ, 嵐にしやがれ, vs 嵐..					
06.05	Male Pop Star - ”Heysayjump”	157,360	1,598,681	2.412	825
’ymdrin_930509’, ’0u0_lso’, ’ichigocherryuki’, ’wolpuu_official’, ’info_hsj’, ’semio_tvasahi’, ’moca622’..					
Jump, Inoo, Arioka, Chinen, hey, Ryosuke Yamada, HiroshiSho, heysayjump, Yamada-kun, nhk Rajira, Tobikko..					
jump, 伊野尾, 有岡, 知念, hey, 山田涼介, 裕翔, heysayjump, 山田くん, nhk らじらー, とびっ子, sa..					
06.06	Male Pop Star - Manzai	128,893	766,044	2.282	538
’matsu_bouzu’, ’owarai_natalie’, ’jinnai_tomonori’, ’kanechi_monster’, ’rinnxofficial’, ’motohage’, ’hen..					
Beef, comic, entertainer, neta, exit, the curtain, comedy, starring, marbled morning star, doors open, c..					
和牛, 漫才, 芸人, ネタ, exit, 開演, お笑い, 出演, 霜降り明星, 開場, コント, 配信中, ゲスト, 放送, 川西, ..					
06.07	Male Pop Star - ”Nissy”	108,859	824,078	2.548	411
’NissyStaff’, ’SHUUTY_ss’, ’SkyHidaka’, ’A_Shinjirooooo’, ’AAA_staff’, ’uno_uno_0716’, ’anation_navi’, ’..					
Nissy, aaa, ice, da, Takahiro Nishijima, Uno, nerd, sky, entertainment, tour, Shuta, live, performances,..					
nissy, aaa, ice, da, 西島隆弘, 宇野, ヲタ, sky, entertainment, tour, 秀太, live..					
06.08	Male Pop Star - ”Kanjani Eight ”	98,900	886,156	2.210	569
’RyoNishikido_JP’, ’subaru_official’, ’takahashiyu’, ’TOWER_Oshikatsu’, ’xxmugendaixx’, ’ryonsari’, ’km0..					
Kanjani, Okura, Subaru, Ryo-chan, Nishikido, Yokoyama, Murakami, Ryo Nishikido, eighter, round-chan, Eig..					
関ジャニ, 大倉, すばる, 亮ちゃん, 錦戸, 横山, 村上, 錦戸亮, eighter, 丸ちゃん, エイト, 安田, 丸山隆平, 安..					
06.09	Male Pop Star - ”Johnny’s West”	90,111	928,852	2.323	732
’_paravi_’, ’jg83220’, ’sg____mg’, ’rysi_fji’, ’sd_kt826’, ’papaJohnnysWEST’, ’otenkinori’, ’kmym___23’, ’..					
Johnny’s west, Shigeoka, west, small Taki, AtsushiFutoshi, Terushi, Hamada, jazz people, meteor, westv, ..					
ジャニーズ west, 重岡, west, 小瀧, 淳太, 照史, 濵田, ジャス民, 流星, westv, 神山, 神ちゃん, 桐山, ジ..					
06.10	Male Pop Star - ”SexyZone”	87,288	1,027,227	2.146	1,502
’bla_kou2019’, ’anan_mag’, ’reco_oshirase’, ’ponycanyon’, ’Shooori_1O3O’, ’chocolate_galbo’, ’nhk_Etele’..					
Sexyzone, Kento, KazeMigaku, sexy, Sekuzo, Katsutoshi Sato, Nakajima, giraffe, victory, black school rul..					
sexyzone, 健人, 風磨, sexy, セクゾ, 佐藤勝利, 中島, 麒麟, 勝利, ブラック校則, 中島健人, 菊池, page..					
06.11	Male Pop Star - ”Bullet Train”	85,646	712,877	2.662	837
’natalie_mu’, ’9taro_cubers’, ’NYLONJAPAN’, ’FINEBOYS_JP’, ’sd_bt’, ’hmvbookssinsaib’, ’iam1101’, ’OKMus..					
Super express, Yuki, Supadora, Takashi, dish, surpassing, Yusuke, milk, ebidan, 8 issue, Takumiumi, lk, ..					
超特急, ユーキ, スパドラ, タカシ, dish, リョウガ, ユースケ, milk, ebidan, 8 号, 匠海, lk, タクヤ,..					
06.12	Male Pop Star - ”News”	74,758	997,379	2.185	1,287
’gochan_V’, ’newsnikoishite’, ’baguette_ntv’, ’dvlgy’, ’ntvsorajiro’, ’voice_ntv’, ’TOKYOMX’, ’moezo_ms’..					
News, Tegoshi, Masuda, worldista, Top Gun, Massu, Takahisa Masuda, Koyama, Yuya Tegoshi, Shige, Shigeaki..					
news, 手越, 増田, worldista, トップガン, まっすー, 増田貴久, 小山, 手越祐也, シゲ, 加藤シゲアキ, 小山慶..					
06.13	Male Pop Star - ”Kis-My-Ft2”	74,575	1,407,830	2.162	1,910
’nhk_musicjp’, ’asadesu0600’, ’jumandekirukana’, ’1242_PR’, ’avexYouTube’, ’avex_portal’, ’paralove_movi..					
Kisumai, kis, ft2, Kitayama, Hiromitsu Kitayama, Yuta Tamamori, my, Tora-san, kismyft, taisuke fujigaya,..					
キスマイ, kis, ft2, 北山, 北山宏光, 玉森裕太, my, トラさん, kismyft, 藤ヶ谷太輔, 横尾, 森くん, 宮田..					
06.14	Male Pop Star - ”Delusion”	71,839	337,719	4.043	1,187
’killerluvv’, ’subako_ysd_love’, ’S_hirano___’, ’hiranoShoKing___’, ’Ry_o___N_’, ’subasibu_0922’, ’MT_JUN_0..					
Nr, pick-up, fufu, love letter, letter, private room, reaction, popular, all right, love, love, EmiEmi, ..					
nr, お迎え, ふふ, ラブレター, letter, 個室, 反応, 大人気, 大丈夫, 好き, love, 笑笑, 浮上, ほんま, ..					
06.15	Male Pop Star - ”Kinkikids”	55,823	833,023	3.089	1,249
’TVguidePERSON’, ’nikkan_entame’, ’teretoongakusai’, ’Daigo19780408’, ’Hochi_Geino’, ’pain_ame’, ’shimad..					
Kinki, kinkikids, Koichi, abcz, black, Tsuyoshi Domoto, v6, Koichi Domoto, light of signs, endrecheri, J..					
kinki, kinkikids, 光一, abcz, black, 堂本剛, v6, 堂本光一, 光の気配, endrecheri, ジ..					
06.16	Male Pop Star - ”Takarazuka”	51,367	521,701	2.324	578

'stage_natalie', 'U_and_YOU', 'toho_stage', 'enterstage_jp', 'spice_stage', 'wowow_stage', 'l_tike_stage..					
Takarazuka, musical, performances, flower set, theater, star sets, Yukigumi, Chu set, the stage, the moo..					
宝塚, ミュージカル, 公演, 花組, 観劇, 星組, 雪組, 宙組, 舞台, 月組, 千秋楽, エリザベート, 井上芳雄, 明日海りお,..					
06.99	Male Pop Star - Others	270,887	2,321,672	2.553	500
'ossans_love', 'anaban_ntv', 'gen_senden', 'Jin_Akanishi', 'jw____jj', 'TOWER_Shinjuku', 'sshota0227', 'd..					
Uncle not a Love, Kazuya Kamenashi, Tomohisa Yamashita, Miura Daichi, Kamenashi, kat, tun, mountain p, s..					
おっさんずラブ, 亀梨和也, 山下智久, 三浦大知, 亀梨, kat, tun, 山 p, 副業, 星野源, ボイメン, dapump, 中..					
07.00	Sexual - FWB(non realistic)	389,298	1,243,799	2.675	242
'sakurakopalmtre', 'okawariema', 'kinokonoosiru', 'onikuosaka7', 'kaorukoosiru', 'lovelessntr', 'rinasci..					
Saffle, recruiting, Ofupako, women, erotic, Ona, Prof, etch, line, Masturbation, virgin, line, Raburitsu..					
セフレ, 募集, オフパコ, 女子, エロ, オナ, プロフ, エッチ, line, オナニー, 童貞, ライン, らぶりつ, 出会い, ..					
07.01	Sexual - mentally ill	325,837	2,126,005	2.957	1,244
'jg86d', 'Meutann15', 'Love_PersonNo1', 'REI_KGM', 'Ione_zest', 'vpw', 'Wa3yMs7u', 'Byr3', 'Li____________s..					
Sick plaque, Forum, the day before ratio, peing, questions, like, Raburitsu, handsome, anonymous, okay, ..					
病み垢, 質問箱, 前日比, peing, 質問, 好き, らぶりつ, 美男, 匿名, 大丈夫, 彼氏, 友達, 募集中, ww, 笑笑,..					
07.02	Sexual - Adult movie	299,812	1,201,943	2.088	344
'av_tamashii', 'kein_yarisugi', 'adaruto30douga', 'arts3204', 'JapaneseAVBabes', 'BoobsTitsOppai', 'denm..					
Erotic, videos, big tits, amateur, tits, cum, beautiful woman, Blow, women, erotic, sex, Gonzo, Masturba..					
エロ, 動画, 巨乳, 素人, おっぱい, 中出し, 美女, フェラ, 女子, エロい, セックス, ハメ撮り, オナニー, 画像, av..					
07.03	-	274,948	1,295,888	2.322	409
'jinghuahuicui', 'Mcgradylsp', 'aiaizhibo', 'nidexiaomugou', 'dagaier888', '69gongshe', '24kingwang', 'b..					
-..					
⊠⊠, ⊠炮, 福利, 微信, 老婆, 美女, 露出, 老公, 自慰, ⊠个, 自己, ⊠⊠, ⊠定, ⊠⊠, ⊠⊠, ⊠免, 妹子, ⊠..					
07.04	Sexual - FWB(young)	245,409	767,234	3.574	467
'ao_hitoto04', 'ma___yu00', '__________asn', 'ugokuonahochaaa', 'ufjk0344', 'xxxx268', 'qmeronn3', 'iam_soa_..					
Women's, men's, dm, erotic, Raburitsu, Ofupako, Saffle, red, recruiting, Ona, Masturbation, tits, etch, ..					
女子, 男子, dm, エロ, らぶりつ, オフパコ, セフレ, アカ, 募集, オナ, オナニー, おっぱい, エッチ, 動画, 質問箱..					
07.05	Sexual - FWB(adult)	244,457	1,550,090	3.292	511
'lov_boscat', 'ikuogakuruo', 'mi0_maniac', 'sersa_h', 'nokochan1224', 'sakisaki_hh', 'r_owa_', 'nanapiii..					
Sex, Forum, love, peing, women, tits, question, work, dm, women, etch, erotic, Saffle, boyfriend, partne..					
セックス, 質問箱, 好き, peing, 女子, おっぱい, 質問, 仕事, dm, 女性, エッチ, エロ, セフレ, 彼氏, 相手,..					
07.06	Sexual - Phone sex	59,134	507,382	2.986	2,650
'harukun0222_', 'pietoro21', 'nextwwwwww', 'RySqt_', 'oQ6_7', 'pure_gumin', 'maru_maruko15', 'TLn874dpJLs..					
Call, sample voice, fell asleep, Mobare, Forum, the plow, the other party, in recruiting, peing, ww, recr..					
通話, サンプルボイス, 寝落ち, モバレ, 質問箱, すき, 相手, 募集中, peing, ww, 募集, 好き, skype, 質問..					
07.07	Sexual - Cross-dressing(as female)	57,309	364,381	3.266	929
'Reneapet57', 'yaoi_trap_bot', 'pigmananal', 'r18pipipi', 'babonpou', 'transexjapan', 'nougami_16344', '..					
Transvestite, transvestite men, Josoko, Transsexual, ⊠娘, shemale, ladyboy, crossdresser, video, ⊠装, Foru..					
女装, 女装男子, 女装子, ニューハーフ, ⊠娘, shemale, ladyboy, crossdresser, 動画, ⊠装, 質問..					
07.08	Sexual - Cross-dressing(as male)	57,112	268,503	3.711	870
'eitohara', 'wolfgang0412', 'kit_channel', 'u_harada', 'ar68241', 'nomiyaaaa127', 'kesp_f', 'nagisa_2524..					
Forum, peing, questions, like, Sekumai, anonymous, lgbt, Tipushi, reaction, tag, friends, work, lgbtq, f..					
質問箱, peing, 質問, 好き, セクマイ, 匿名, lgbt, ティプシー, 反応, タグ, 友達, 仕事, lgbtq, fre..					
07.99	Sexual - Others	267,922	1,065,151	3.012	333
'100patu100cyu', 'KTG_wo_ka', 'Shibarijp', 'meroqiqi', 'Hirugao_69', 'Zoikhem1', 'ex_fuck_off', 'chocomi..					
Masturbation, erotic, boyfriend, Saffle, line, Forum, women's, videos, like, recruiting, Ofupako, peing,..					
オナニー, エロ, 彼氏, セフレ, line, 質問箱, 女子, 動画, 好き, 募集, オフパコ, peing, 女性, おっぱい, ..					
08.00	Game(femele) - "Demon Slayer"	356,409	3,414,943	3.020	415
'kimetsu_off', 'avogado6', 'ufotable', 'heroaca_anime', 'horikoshiko', 'animehaikyu_com', 'ryosuketarou'..					
Blade of Onimetsu, dec, Jiro, like, Hiroaka, My Hero Academia, briquettes, cartoon, Haikyu, illustration..					
鬼滅の刃, デク, 治郎, 好き, ヒロアカ, 僕のヒーローアカデミア, 煉炭, 漫画, ハイキュー, イラスト, パロ, 本誌, 煉獄,..					
08.01	Game(femele) - "Touken Ranbu"	351,146	3,570,263	1.957	496
'hanae0626', 'KAJI_staff', 'conan_movie', 'animatetimes', 'mankai_company', 'Anime_AbemaTV', 'magazine_p..					
Events, public, performances, play at night, broadcast, sale, voice, gifts, fun, everyone, information, ..					

イベント, 公開, 公演, 夜あそび, 放送, 発売, 声優, プレゼント, 楽しみ, 皆様, 情報, cv, 開催, 出演, 登場, 来..					
08.02	Game(femele) - marketplace	238,287	2,916,408	5.874	838
'yume335', 'coade_official', 'comicBUNKO', 'chan_shiori', 'tokires_info', 'conazeraci', 'jumpfesta', '44..					
Exchange, transfer, you can badge, mailing, please, handed, shipping, possible, find, price, review, tra..					
交換, 譲渡, 缶バッジ, 郵送, お願い, 手渡し, 送料, 可能, 検索, 定価, 検討, 取引, お返事, 失礼, 譲り, 申し訳,..					
08.03	Game(femele) - "Hypnosis Mic"	139,634	2,146,964	1.935	688
'hypnosismic', 'GiantSUBAru', 'hypnosismic_arb', 'marui_anime', 'komaDwataru', 's_kamio113', 'nozuyama12..					
Hipumai, hypnosis microphone, the magnetic path, ambulatory, love, division, sub, Hod, Hifumi, battle, r..					
ヒプマイ, ヒプノシスマイク, じろ, 独歩, 好き, ディビジョン, さぶ, ふど, ひふみ, battle, 乱数, 先生, tdd,..					
08.04	Game(femele) - "Ensemble Stars"	132,380	1,381,480	2.080	769
'ensemble_stars', 'stars_animation', 'DPInc_official', 'ensemble_stage', 'enstars_music', 'last_period',..					
Ansuta, static, ensemble Stars, Leo, scout, events, Sutarai, publishing, knights, meteor Corps, love, un..					
あんスタ, スタ, あんさんぶるスターズ, レオ, スカウト, イベント, スタライ, 公開, knights, 流星隊, 好き, ユニッ..					
08.05	Game(femele) - Cosplay	121,789	1,398,615	3.040	351
'risingbeat_app', 'shintenianime', 'airilyweb', 'comiclandjp', 'assist_wig', 'a3kouryaku', 'swallow_tail..					
Kos, Cosplay, a3, layers, photo, costumes, love, cosplay, photography, wig, photos, events, appointments..					
コス, コスプレ, a3, レイヤー, photo, 衣装, 好き, cosplay, 撮影, ウィッグ, 写真, イベント, 予定, プ..					
08.06	Game(femele) - Illustration	102,517	632,464	3.534	499
'jin_jin_suruyo', 'StudioWasabi_', 'ibisPaint', 'nannkizum', 'strk_tenshi', 'KaNeN___', 'mihiro_00122', '..					
Drawing, illustration, creative, love, cluster, character, design, analog, painter, painting, graffiti, ..					
絵描き, イラスト, 創作, 好き, クラスタ, キャラ, 絵柄, アナログ, 絵師, お絵描き, 落書き, 関係, 企画, 質問箱, タ..					
08.07	Game(femele) - "Idolish7"	87,098	1,203,261	1.960	752
'iD7Mng_Ogami', 'ichibanKUJI', 'arinacchi', 'banpreikebukuro', 'yuhiro4949', 'ID7_anime', 'gashaponanime..					
Ainana, eyed Irish Seven, Nana, eye, idolish7, one woven, trigger, Yamato, Nagi, reunion, źooļ, vale, co..					
アイナナ, アイドリッシュセブン, ナナ, アイ, idolish7, 一織, trigger, 大和, ナギ, reunion, źoo..					
08.08	Game(femele) - "Naruto"	86,139	255,307	2.092	457
'hiro_mashima', 'mega_girlshobby', 'fairytail_PR', 'szmallow_xx', 'NARUTOtoBORUTO', 'panako2557', 'Hunte..					
Naruto, naruto, Sasuke, Sakura, Kakashi, Sasusaku, Natsu, boruto, Obito, bleach, fairy tail, the, teache..					
ナルト, naruto, サスケ, サクラ, カカシ, サスサク, ナツ, boruto, オビト, bleach, フェアリーテイル, ..					
08.09	Game(femele) - "Uta no Prince-sama"	71,149	831,279	2.231	1,358
'utapri_official', 'agematsu', 'shininglive_jp', 'LaRicetta99', 'HEAVENS_staff', 'Tokiya_I_SH', 'Ren_J_S..					
Song Puri, exchange, Oto 也, Tokiya, Ren, Mato, Kingdom, Camus, of the song, Tsu Prince-sama, Cecil, Ranma..					
うたプリ, 交換, 音也, トキヤ, レン, 真斗, キングダム, カミュ, うたの, プリンスさまっ, セシル, 蘭丸, 那月, 缶バッ..					
08.10	Game(femele) - Yaoi	65,007	400,856	2.648	709
'sakurabihashigo', 'morisita_suu', 'chillchill_bl', 'dear_plus_', 'harada_info', 'tyottokame', 'b_szmc',..					
Teacher, bl, sale, love, manga, yaoi fandom, benefits, Comics, rot boys, new book, animation, position, ..					
先生, bl, 発売, 好き, 漫画, 腐女子, 特典, コミックス, 腐男子, 新刊, アニメ, いち, 作品, アニメイト, 配信, ..					
08.11	Game(femele) - "Bungo Stray Dogs"	52,432	494,976	2.078	1,070
'bungosd_anime', 'bungomayoi', 'aka3kan', 'sayamaru_417', 'bungo_stage', 'bungostraydogs', 'bungosd_sanr..					
Among writer Stray Dogs, strike, bold, Dazai, Ju, bungosd, love, Chuya, illustrations, exceptionally tal..					
文豪ストレイドッグス, スト, 太中, 太宰, ちゅ, bungosd, 好き, 中也, イラスト, 異能, ドス, アニメ, ssr, ..					
08.99	Game(femele) - Others	405,486	3,544,807	2.914	347
'cinnamon_sanrio', 'sanrio_news', 'purin_sanrio', 'osomatsu_movie', 'Melody_Mariland', 'anime_shingeki',..					
Love, Mr. Osomatsu, larch, character, Osomatsu's movie, Osomatsu, illustration, animation, Hitotsumatsu,..					
好き, おそ松さん, カラ松, キャラ, えいがのおそ松さん, おそ松, イラスト, アニメ, 一松, お願い, イベント, 銀魂, カラ..					
09.00	Sports/etc - funny short clip	441,675	1,258,670	2.542	310
'omosiro_geki', 'Twitube_123', 'kokoro_odoru_1', 'omosirooidouga', 'kusowaraeruwwww', 'joker_budou', 'or..					
Forum, peing, question, laugh, anonymous, in recruiting, EmiEmi, fresh, box, www, like, wwww, www, boyfr..					
質問箱, peing, 質問, 爆笑, 匿名, 募集中, 笑笑, fresh, box, www, 好き, wwww, ｗｗｗ, 彼氏, ..					
09.01	Sports/etc - Football	266,673	2,331,695	2.240	715
'YutoNagatomo5', 'J_League', 'kskgroup2017', 'DAZN_JPN', 'SoccerKingJP', 'GoalJP_Official', 'gekisaka', ..					
Players, soccer, game, j league, goal, j league, vs, team, club, director, supporters, Urawa, Kashima, S..					
選手, サッカー, 試合, j リーグ, ゴール, ｊリーグ, vs, チーム, クラブ, 監督, サポーター, 浦和, 鹿島, スタジアム..					
09.02	Sports/etc - Baseball	188,106	1,902,847	2.296	816

'faridyu', 'gnomotoke', 'sn_baseball_jp', 'TigersDreamlink', 'FightersPR', 'npb', 'fukudasun', 'Pacificl..					
Players, pitcher, Hanshin, baseball, baystars, China and Japan, the game, Yakult, baseball team, npb, Or..					
選手, 投手, 阪神, 野球, baystars, 中日, 試合, ヤクルト, 球団, npb, オリックス, ホームラン, seibul..					
09.03	Sports/etc - Basketball	101,072	580,213	2.322	980
'ct_nba', 'NBAJPN', 'B_LEAGUE', 'JAPANBASKETBALL', 'BASKETLIVE_JP', 'bbking_jp', 'nbabot3..					
Players, b league, basketball, nba, game, team, vs, cheering, basketball, play, basket, shoot, season, A..					
選手, b リーグ, バスケ, nba, 試合, チーム, vs, 応援, バスケットボール, プレー, バスケット, シュート, シーズン..					
09.04	Sports/etc - Motorsport culture	99,634	190,681	4.111	475
'____sho_ma', 'sk391023', 'danjiri_ohan', '47Chimu2', 'Teru7890Teru', 'dic_key_dik', 'Hokuto004240', 'RM1..					
Laughter, Forum, Honma, peing, questions, EmiEmi, anonymous, in recruiting, fresh, box, work, Danjiri, H..					
爆笑, 質問箱, ほんま, peing, 質問, 笑笑, 匿名, 募集中, fresh, box, 仕事, だんじり, 本間, プレゼント..					
09.05	Sports/etc - Motorsport	93,746	354,623	3.223	1,342
'SlideJPN', 'yakara17vx', 'xdocomo666', 'kurodoradora', 'selecter_seiji', 'Sexy_President', 'ryugrs19', ..					
Forum, EmiEmi, peing, wheel, questions, ww, photo, work, tire, compared with the day before, genuine, ca..					
質問箱, 笑笑, peing, ホイール, 質問, ww, 写真, 仕事, タイヤ, 前日比, 純正, 洗車, mt, 匿名, 募集中, ..					
09.06	Sports/etc - remodeled car.	76,956	196,804	3.075	1,507
'shochaserti', 'Jason_fuckinJPN', '86_8chaaaamao', 'ANIMALSTYLE_J', 'junjunmaru67', 'issei0418', 'Celsio..					
Forum, peing, EmiEmi, question, anonymous, in recruiting, Sapporo, fresh, answer, Seriously, work, laugh..					
質問箱, peing, 笑笑, 質問, 匿名, 募集中, 札幌, fresh, 回答, まじ, 仕事, 爆笑, box, 好き, 北海道,..					
09.07	Sports/etc - Marathon	71,268	262,667	2.643	746
'jaaf_official', 'sugurusako', 'kashi0713', 'EKIDEN_News', 'ekiden_mania', '4years_media', 'ushirokeisuk..					
Players, run, nice, marathon, land, men, Hakone Ekiden, Ekiden, Forum, practice, tournament, peing, race..					
選手, ラン, ナイス, マラソン, 陸上, 男子, 箱根駅伝, 駅伝, 質問箱, 練習, 大会, peing, レース, 応援, ランニ..					
09.08	Sports/etc - highschool baseball	60,940	190,067	2.405	838
'asahi_koshien', 'kazutan_1220', 'gari_wasabi', '_F_8_9_', 'koshienasahi', 'nhk_koushien', 'nikkan_tohok..					
High school baseball, baseball, players, Koshien, tournament, pitcher, Spring, game, Osakatoin, high sch..					
高校野球, 野球, 選手, 甲子園, 大会, 投手, 春季, 試合, 大阪桐蔭, 高校, 秋季, 星稜, 吉田輝星, 習志野, 監督, セ..					
09.09	Sports/etc - football game("WE")	57,347	404,559	3.181	1,390
'hikacgames', 'we_konami', 'chamakunchi', 'pes60130924', 'messu_009', '_WiNNIng_1_1', 'volante_info', 'd..					
Uiire, Uiire app, players, participation, fp, alliance, competition, hope, hope, EmiEmi, Furema, descrip..					
ウイイレ, ウイイレアプリ, 選手, 参加, fp, 同盟, 大会, 希望, お願い, 笑笑, フレマ, リプ, itunes, サッカー..					
09.10	Sports/etc - football game("FIFA")	56,713	198,405	2.584	677
'Arsenal', 'FCBarcelona', 'EdWoodwardGod', 'yuugooner', 'goal', 'ogawa3313', 'realmadrid', 'juventusfcjp..					
Athletes, fifa, game, Arsenal, club, Barca, Chelsea, Madrid, soccer, united, team, Messi, Transfers, goa..					
選手, fifa, 試合, アーセナル, クラブ, バルサ, チェルシー, マドリー, サッカー, ユナイテッド, チーム, メッシ, 移..					
09.11	Sports/etc - Baseball("Giants")	52,288	298,521	2.454	999
'TokyoGiants', 'hochi_giants', 'ntv_baseball', 'TeamUehara', 'pawapuro_pro', 'wed_downtown', 'sanspo_gia..					
Giants, Giants, giants, players, pitcher, Purosupi, Seiji Kobayashi, Sakamoto, Hayato Sakamoto, Okamoto,..					
巨人, ジャイアンツ, giants, 選手, 投手, プロスピ, 小林誠司, 坂本, 坂本勇人, 岡本, 小林, 菅野, 一軍, ホーム..					
09.99	Sports/etc - Others	419,404	1,653,348	2.751	408
'rikakoikee', 'HAWKS_official', 'FOXSportsJP', 'chugoku_carp', 'aifukuhara1101', 'S_Kagawa0317', 'tbc_mo..					
Players, Carp, sbhawks, carp, Forum, peing, darts, games, table tennis, questions, Hiroshima, Hawks, che..					
選手, カープ, sbhawks, carp, 質問箱, peing, ダーツ, 試合, 卓球, 質問, 広島, ホークス, 応援, 笑笑..					
10.00	-	175,126	227,399	1.726	26
'QuebrandoOTabu', 'LwithP', 'luscas', 'arctmankeys', 'fuckyeahluke', 'craiskc', 'dougraz', 'NetflixBrasi..					
-..					
ｍｅｓｔｒｅ, de, que, eu, não, do, da, pra, essa, minha, um, ｓｉｍ, você, ｔｅ..					
10.01	-	115,532	144,958	1.690	5
'el25villero', 'elcosodelapizza', 'salvavidxs', 'Agustinslzr', 'PrototypePIayer', 'agusalejook', 'louand..					
-..					
de, que, en, no, me, mi, la, los, te, el, pero, hola, quiero, mucho, ..					
10.02	-	82,333	98,042	1.956	35
'9GAG', 'ulat_bulu_bulu', 'HanifHaiqalll', 'takaharasuiko', 'pja_chan', 'sunfloweraidil', 'Alhamdhulilla..					
-..					

you, me, the, is, my, when, and, this, it, in, that, your, if, of, do..					
10.03	-	77,189	96,157	1.710	57
’japonesaldia’, ’SadDazz’, ’colourwxve’, ’illoquepereza’, ’ortograconejito’, ’kike_g7’, ’MHerespa’, ’alv..					
-..					
de, que, el, la, no, en, es, los, si, te, por, me, con, las, un, una,..					
10.04	-	65,979	78,914	1.757	22
’evianFrance’, ’RebeuDeter’, ’db_0528’, ’margielasaint’, ’LoosGuccreen’, ’SaadBNK’, ’MajidTentacion’, ’l..					
-..					
est, de, les, le, la, pas, je, ça, un, et, des, que, qui, pour, vous,..					
10.05	-	56,268	62,785	1.896	23
’MuslimIQ’, ’QasimRashid’, ’Joshua4Congress’, ’IlhanMN’, ’RonWyden’, ’MillennialProf_’, ’Ishikawa_Sachi’..					
-..					
the, is, and, of, repost, it, you, that, this, for, in, not, with, my..					
10.06	-	51,753	54,828	1.650	4
’sincerojesuis’, ’memeinutil’, ’ErrosDosJovens’, ’trouxavcs’, ’yr_lobo’, ’umameninasolta’, ’euotrouxa’, ..					
-..					
minha, anos, últimos, nos, eu, c a r e n t e, paciência, de, m a s, não, quer..					
10.99	-	1,329,731	1,767,956	1.977	22
’ArianaGrande’, ’ogecebel’, ’yousannnn’, ’bunnyarchive’, ’Jerrypleasure’, ’NihongoSOS’, ’carIys’, ’Ridic..					
-..					
the, you, and, ゲスト, me, my, ゜゜, de, is, this, of, in, for, repost, it..					
11.00	Politics/etc - Art	378,335	2,416,884	3.013	408
’amass_jp’, ’utadahikaru’, ’WORLDJAPAN’, ’69_meigen’, ’Yano_Akiko’, ’K_Onishi’, ’otomojamjam’, ’michinar..					
The, movie, dj, music, work, band, performer, Japan, held, lowest price, supervision, amp, Tokyo, open, ..					
the, 映画, dj, 音楽, 作品, バンド, 出演, 日本, 開催, 最安値, 監督, amp, 東京, open, 上映, 予約,..					
11.01	Politics/etc - Right echo-chamber	269,791	9,266,349	1.997	2,002
’Sankei_news’, ’konotarogomame’, ’chowtingagnes’, ’katsuyatakasu’, ’AbeShinzo’, ’anonymous201504’, ’hyak..					
South Korea, Japan, members, China, Japan, the opposition, the people, North Korea, problems, criticism,..					
韓国, 日本, 議員, 中国, 日本人, 野党, 国民, 北朝鮮, 問題, 批判, ネット, 韓国人, 共産党, 報道, マスコミ, 反日..					
11.02	Politics/etc - Left echo-chamber	251,036	13,371,458	1.944	3,533
’mainichi’, ’kyodo_official’, ’kikko_no_blog’, ’jijicom’, ’TomoMachi’, ’asahi’, ’emorikousuke’, ’HuffPos..					
Japan, Abe, the people, the Liberal Democratic Party, Abe administration, lawmakers, Prime Minister Shin..					
日本, 安倍, 国民, 自民党, 安倍政権, 議員, 安倍首相, 山本太郎, 問題, 野党, 政権, 国会, 政治, 政府, 辺野古, 消..					
11.03	Politics/etc - Mental health	174,215	1,234,957	3.383	694
’TsujiHitonari’, ’kagekineko’, ’_miwa_akihiro’, ’Hirohina6’, ’enicat10’, ’haba_survivor’, ’tatakau_sangy..					
Children, children, quote, work, problems, needs, human beings, words, developmental disabilities, schoo..					
子ども, 子供, 名言, 仕事, 問題, 必要, 人間, 言葉, 発達障害, 学校, 先生, 日本, meigen, 人生, 相手, 介護..					
11.04	Politics/etc - cryptocurrency	141,845	888,802	2.624	1,011
’nikkei’, ’Reuters_co_jp’, ’hiroyuki_ni’, ’BloombergJapan’, ’Toyokeizai’, ’okasanman’, ’WSJJapan’, ’tsub..					
Virtual currency, Bitcoin, investment, fx, btc, xrp, stocks, market, dollar yen, news, Japan, ripple, tr..					
仮想通貨, ビットコイン, 投資, fx, btc, xrp, 銘柄, 相場, ドル円, ニュース, 日本, リップル, トレード, 中国..					
11.05	Politics/etc - motorcycle touring	123,883	1,518,615	3.601	1,005
’PutiMotor’, ’antibcsc’, ’whitebase1’, ’daytona675675’, ’rikitauko’, ’motegi_official’, ’wokarider’, ’ta..					
Bike, touring, rider, Forum, delivered, peing, models, in, at, EmiEmi, gender, the day before ratio, age..					
バイク, ツーリング, ライダー, 質問箱, 納車, peing, 車種, in, at, 笑笑, 性別, 前日比, 年齢, 身長, 写真..					
11.06	Politics/etc - Disaster news	66,979	257,178	2.202	733
’rugbyworldcupjp’, ’sosotakei’, ’YahooTopicsEdit’, ’BosaiNaganoPref’, ’JRFUMedia’, ’chiba3ch’, ’ynwataig..					
Rugby, player, rwc, recording, information, disaster, occurrence, Japan, news, fire, map, New Komeito, w..					
ラグビー, 選手, rwc, 録画, 情報, 災害, 発生, 日本, ニュース, 火事, まっぷ, 公明党, ドコ, 救急, 支援, 千葉..					
11.07	Politics/etc - sex workers	58,595	316,672	3.040	779
’irohasujo_’, ’mamamasakiss’, ’bakausapipi’, ’rie0985’, ’shibu_natsu’, ’yoro_sh1ku’, ’aoi_hinano’, ’miyu..					
Host, migrant, guarantee, responsible, customs, scout, nomination, Kabukicho, Forum, attendance, girls, ..					
ホスト, 出稼ぎ, 保証, 担当, 風俗, スカウト, 指名, 歌舞伎町, 質問箱, 出勤, 女の子, 在籍, 優木, お金, peing..					
11.08	Politics/etc - Classic music	56,190	232,142	2.834	773

'sendaiphil', 'AsahiBrass', 'musique_bot', 'japan_arts', 'kanagawaphil', '880hz', 'kyohei0901', 'chisako..					
Playing, piano, music, concert, musical instruments, concert, brain Bell show, practice, conductor, bras..					
演奏, ピアノ, 音楽, コンサート, 楽器, 演奏会, 脳ベル show, 練習, 指揮, 吹奏楽, レッスン, 楽譜, クラシック, 先..					
11.09	-	51,264	66,383	2.216	141
'realDonaldTrump', 'narendramodi', 'DLoesch', '178kakapo', 'NHKWORLD_News', 'alfonslopeztena', 'BarackOb..					
-..					
the, of, and, in, is, for, that, you, on, this, it, trump, are, with,..					
11.99	Politics/etc - Others	206,608	1,096,702	3.508	379
'tenkijp_jishin', 'sputnik_jp', 'gjmorley', 'sumokyokai', 'nodasori2525', 'ogiriking', 'aart_bot', 'Ichi..					
Code, Japan, day luck, earthquake, nhk, need, Amagasemachi, English, population stalker, problem, man, w..					
コード, 日本, 日運, 地震, nhk, 必要, 天瀬, 英語, 集団ストーカー, 問題, 人間, 仕事, 占い, 女性, 創価学会, ..					
12.00	Online Game - "Fortnite"	296,766	2,718,432	2.511	1,490
'FortniteJP', 'ara_to1', 'VodkaChaso', '___Necko', 'NK_aniki', 'tenchim_1119', 'urara_123urara', 'Osamint..					
Fortnight, fortnite, ps4, compared with the day before, Fotona, participation, Forum, share, kill, pleas..					
フォートナイト, fortnite, ps4, 前日比, フォトナ, 参加, 質問箱, share, キル, お願い, 配信, 質問, ゲ..					
12.01	Online Game - "splatoon"	227,197	2,601,157	3.249	1,508
'SplatoonJP', 'hanjyou_', 'ika_shicomcom', 'taijich0324', 'NPB_esports_spl', '3RNcake_L', 'moronnoseidab..					
Splatoon 2, splatoon2, nintendo, switch, Riguma, spline, Forum, peing, Purabe, turf, squid, Gachima, kil..					
スプラトゥーン 2, splatoon2, nintendo, switch, リグマ, スプラ, 質問箱, peing, プラベ, ナワバ..					
12.02	Online Game - "Rainbow Six Siege"	161,154	1,423,470	2.715	1,283
'nakanocchi2', 'Rainbow6JP', 'simonetaB0T', 'SiegeChannel', 'norarengou', 'UBISOFT_JAPAN', 'RqyIeigh', '..					
Siege, ps4, rank, share, Forum, Rainbow Six Siege, peing, clan, games, questions, live, pc, Rainbow Six,..					
シージ, ps4, ランク, share, 質問箱, レインボーシックスシージ, peing, クラン, ゲーム, 質問, live, p..					
12.03	Online Game - "PUBG"	142,397	1,155,297	2.900	1,033
'stylishnoob', 'LoLJPOfficial', 'jpPlayOverwatch', 'taisyoupants', 'avashaka', 'fog_912', 'Official_LJL'..					
Pubg, lol, game, apex, Forum, delivery, peing, question, over-watch, pc, ps4, kill, youtube, compared wi..					
pubg, lol, ゲーム, apex, 質問箱, 配信, peing, 質問, オーバーウォッチ, pc, ps4, キル, yout..					
12.04	Online Game - "Monster Hunter"	142,173	1,169,982	3.418	915
'ApexLegendsWiki', 'DeadbyBHVR_JP', 'ApexTimes', 'don_21st', 'ApexLeaks_Marin', 've1cra', 'DbDinformatio..					
Ps4, mhw, Monster Hunter, apex, dbd, share, game, Forum, ice bone, recruiting, peing, distribution, live..					
ps4, mhw, モンハン, apex, dbd, share, ゲーム, 質問箱, アイスボーン, 募集, peing, 配信, li..					
12.05	Online Game - "Final fantasy"	140,541	1,988,249	2.640	1,237
'FF_XIV_JP', 'umadori0726', 'FF14_sokuhou', 'SOKENsquareenix', '298tp', 'halu18', 'kugelcruor_Draw', 'Fr..					
Ff14, ss, Lara, equipment, tanks, jet black, Raraferu, Gila, ps4, gposers, clear, aura, ffxiv, share, ho..					
ff14, ss, ララ, 装備, タンク, 漆黒, ララフェル, ヒラ, ps4, gposers, クリア, アウラ, ffxiv, ..					
12.06	Online Game - playing video	105,580	867,347	3.552	1,366
'EAA_tw', 'omotemaru', 'R9cing', 'CallofDutyJP', 'bulletjda', 'k4sen', 'OMGJKREAL', 'RushGamingJP', 'Nya..					
Bo, ps4, plzbo, please, recruiting, share, cod, game, Ontsu, Forum, live, question, broadcast, black-out..					
bo, ps4, plzbo, お願い, 募集, share, cod, ゲーム, おんつ, 質問箱, live, 質問, ブロードキャス..					
12.07	Online Game - maketplace of items	79,427	562,644	1.866	2,291
'HxDHax', '0z_u_', '247255', 'NAG5c', 'Tsum_Officiall', 'kyle_shop', '987674', 'Sho6_5', 'Axell_FT', '14..					
Alternate, Fortnight, Tsumutsumu, Roman explosion, performance, sale, Tsumutsumukoin, iconic, lottery, dm..					
代行, フォートナイト, ツムツム, フォロ爆, 実績, 販売, ツムツムコイン, アイコニック, 抽選, dm, アカウント, 導入, ..					
12.08	Online Game - "Pokemon"	61,547	314,574	3.308	269
'gaonyan412', 'Realism___1', 'syumiPokemon', 'pokemon_game8', 'Re_mom_', 'kajitetu2072228', 'piro____s', '..					
Recruitment, lv, id, war, Pokemon, exchange, Magna, Metamon, gifts, please, hatching, campaign, differen..					
参加者募集, lv, id, 参戦, ポケモン, 交換, マグナ, メタモン, プレゼント, お願い, 孵化, キャンペーン, 色違い, ..					
12.09	Online Game - "Doragon Quest"	54,156	622,322	3.513	1,390
'DQ_X', 'DQ_RIVALS', 'ayanatsubaki', 'natural9ryu', 'oteu_dq10', 'ani_p_p', 'dq_tora', 'rizarth', 'meir_..					
Dragon Quest 10, dq10, Dragon Quest, Dorea, dqx, equipment, frame, Rivals, Forum, Friends, peing, ww, pu..					
ドラクエ 10, dq10, ドラクエ, ドレア, dqx, 装備, フレ, ライバルズ, 質問箱, フレンド, peing, ww, 討伐..					
12.99	Online Game - Others	274,832	2,068,962	3.621	516
'Otojya', 'netatank', 'HARUTYA1226', 'otsuichich', 'BacchioSnow', 'norun9', 'roadhog_KUN', 'Tettou_', 'D..					
Ps4, games, Forum, peing, videos, questions, live, delivery, the day before ratio, switch, game play-by-..					

ps4, ゲーム, 質問箱, peing, 動画, 質問, live, 配信, 前日比, switch, ゲーム実況, 実況, share..					
13.00	Animated music band - ”Urashimasakatasen”	288,865	3,628,073	2.532	715
’uni_mafumafu’, ’uratasama’, ’_amatsuki_’, ’soraruru’, ’sakatandao’, ’USSS_info’, ’sen_sen_sen_sen’, ’sh..					
Urashima Sakata ship, Mafumafu, Soraru, Shima, Senra, Amatsuki, Sakata, post, singer, videos, tried to s..					
浦島坂田船, まふまふ, そらる, 志麻, センラ, 天月, 坂田, 投稿, 歌い手, 動画, 歌ってみた, グッズ, 失礼, 好き, 楽..					
13.01	Animated music band - ”Strawberry Prince”	280,243	3,804,752	2.389	987
’Jel___official’, ’StPri_info’, ’p_ma_ru’, ’satomimi___’, ’Colon56Nsab’, ’rinu_sub’, ’root_nico_2’, ’rinu_..					
Strike, pre, www, colon, 莉犬, Upotsu, Satomi, and roux, gels, gallery, relay, personality, notes, videos,..					
すと, ぷり, www, ころん, 莉犬, うぽつ, さとみ, るぅと, ジェル, ギャラリー, リレー, 人格, めも, 動画, わこ,..					
13.02	Animated music band - online streaming	205,430	1,070,970	3.542	928
’Q0S_Rimu’, ’kamahen_’, ’argturuinmd’, ’takayan_gorizal’, ’hata_natsuha_’, ’nanashi_lie’, ’Dboy0112’, ’k..					
Forum, peing, Cass, Moi, during the delivery, questions, first look, delivery, ww, anonymous, love, ipho..					
質問箱, peing, キャス, モイ, 配信中, 質問, 初見, 配信, ww, 匿名, 好き, iphone, 雑談, 募集中, ww..					
13.03	Animated music band - music distribution	163,331	1,250,942	3.049	1,077
’nabuna2’, ’SiNxxx526’, ’164203’, ’memememememe28’, ’Umi_babe’, ’tikandame’, ’1code_a’, ’yr_boubou’, ’Sm..					
I tried to sing, Forum, nana, singer, peing, mix, videos, posts, sm, mix nurses, questions, like, Cass, ..					
歌ってみた, 質問箱, nana, 歌い手, peing, mix, 動画, 投稿, sm, mix 師, 質問, 好き, キャス, ww,..					
13.04	Animated music band - ”MineCraft”	113,549	1,348,753	2.438	909
’oowareware1945’, ’natukasii_sekai’, ’hakaiGia’, ’tomo0723sw’, ’radaokun’, ’p1kt3’, ’peintooon’, ’wrwrdz..					
Wrwrd, love, video, Micra, werewolf, Forum, drawing, peing, illustration, post, question, teacher, forge..					
wrwrd, 好き, 動画, マイクラ, 人狼, 質問箱, 絵描き, peing, イラスト, 投稿, 質問, 先生, 捏造, 実況, t..					
13.05	Animated music band - Singing/playing anime song	72,668	627,830	4.237	940
’kurokumo_01’, ’sakuya_252525’, ’x_127x’, ’cyuunisakkunn’, ’benymd_bot’, ’osato_sato’, ’Lilonosub’, ’Qpx..					
Plow, called Tame, Sakata, www, Urashima Sakata ship, love, Mafumafu, strike, welcome, love, Forum, pick..					
すき, 呼びタメ, 坂田, www, 浦島坂田船, だいすき, まふまふ, すと, 歓迎, 好き, 質問箱, お迎え, そらる, 歌い手,..					
13.06	Animated music band - ”M.S.S Project”	68,699	593,524	2.798	687
’kiyo_saiore’, ’retokani’, ’kirizaki_ei’, ’Gatchman666’, ’ushizawa’, ’eoheoh_out’, ’fuji_saiore’, ’Pocky..					
Mssp, play-by-play, videos, posts, Kiyo, over over, like, Naples, games, live, illustrations, questions,..					
mssp, 実況, 動画, 投稿, キヨ, ーー, 好き, ナポリ, ゲーム, 生放送, イラスト, 質問, 10 周年, youtube,..					
13.07	Animated music band - ”Korekoi”	68,441	480,804	2.296	557
’korekore19’, ’GC5R5OGIKgV0yvz’, ’korekore_ch’, ’8IcoEitHGybnh5V’, ’ykm9r’, ’fenichan_dayo’, ’Xykmr’, ’V..					
Elapsed, korekore, distribution, uneven, singer, snow, Cass, Korekore, strike, www, Nikokyasu, grasp, cr..					
経過, korekore, 配信, むら, 歌い手, ゆき, キャス, コレコレ, すと, www, ニコキャス, 把握, かに, ぷり,..					
13.99	Animated music band - Others	187,825	1,449,157	2.843	1,019
’shito_stereo’, ’yamako2626’, ’yasuner1103’, ’otaki1207’, ’kooku46’, ’RRReol’, ’ri_ri_ri_5’, ’antsa_www’..					
I tried dancing, Forum, peing, love, mesemoa, questions, ww, videos, lispon, fun, live, sm, EmiEmi, okay..					
踊ってみた, 質問箱, peing, 好き, mesemoa, 質問, ww, 動画, lispon, 楽しみ, live, sm, 笑笑..					
14.00	Mobile Game - ”Knives Out”	238,951	3,770,300	1.992	2,091
’oreratuyoi’, ’MaximusHotoke’, ’S_Bocky’, ’Defeee4’, ’X0X08008’, ’sou6282’, ’ZT_Aegis’, ’Vo1x_’, ’4y_u’,..					
Guerrillas, wilderness behavior, wilderness, duo, prize, kill, confirmed, representative, conditions, pa..					
ゲリラ, 荒野行動, 荒野, デュオ, 賞金, キル, 確定, 代表者, 条件, 参加, クラン, ルーム, 大会, リプ, チーム名, ..					
14.01	Mobile Game - ”Knives Out”(Campaign)	205,477	903,155	1.984	995
’GAME_KNIVES_OUT’, ’tyoumukakin7’, ’hitohito12O’, ’QOQO114514114’, ’ginnan_0’, ’DT43922294’, ’mainiki_____..					
Wilderness action, itunes, wilderness, gift planning, planning, cards, participation, conditions, 1500 y..					
荒野行動, itunes, 荒野, プレゼント企画, 企画, カード, 参加, 条件, 1500 円, オン, プレゼント, 通知, リプ,..					
14.02	Mobile Game - ”Monster Strike”	85,162	732,286	3.092	1,540
’___t___c’, ’shiro_gw’, ’gangimari_tarou’, ’nupl_gw’, ’xxxstnu’, ’monst_riex’, ’yuumis215’, ’mnst_kd’, ’na..					
Monsuto, circling, beast apotheosis, contraindications, character, coat of arms, multi, Forum, orbs, tem..					
モンスト, 周回, 獣神化, 禁忌, キャラ, 紋章, マルチ, 質問箱, オーブ, 神殿, 至宝, 前日比, ガチャ, ラキモン, お願..					
14.03	Mobile Game - ”Puzzle & Dragons”	73,388	735,269	3.034	2,159
’yuukoru_’, ’shussyussyu’, ’DaikeYamamoto’, ’aheaheeeeeeeee’, ’outsider_39’, ’takenama2525’, ’suhadasuha..					
Pazudora, compared with the day before, Randan, profile, rank, Forum, circling, please, organize, Arenes..					
パズドラ, 前日比, ランダン, 異形, ランク, 質問箱, 周回, お願い, 編成, 闘技, フレンド, 王冠, パズドラー, pein..					
14.04	Mobile Game - ”Clash Royale”	72,823	578,900	3.052	2,278

'brawlstarsjp', 'ClashRoyaleJP', 'kurakurachannel', 'kiokio_coc', 'Kent_Golemeshi', 'isaporonCRb', 'YAPI..					
Kurarowa, Burosuta, clan, Toro, Miratibu, delivery, friend, dizzy, please, Forum, the day before ratio, ..					
クラロワ, ブロスタ, クラン, トロ, ミラティブ, 配信, フレンド, クラクラ, お願い, 質問箱, 前日比, mirrativ, ..					
14.05	Mobile Game - "PUBG"	70,022	717,081	3.098	1,964
'MAGARETSUTV', 'MAGARETONN', 'LHY_LIMITED', 'pnsk_gw', 'Hey_Kun_Kun', 'aotonbo_nico', 'Vo..					
Pubg, mobile, mobile, Don wins, delivery, Forum, peing, scrim, mirrativ, kill, Miratibu, ww, question, p..					
pubg, mobile, モバイル, ドン勝, 配信, 質問箱, peing, スクリム, mirrativ, キル, ミラティブ, w..					
14.99	Mobile Game - Others	417,770	3,651,940	3.858	478
'mirrativ_jp', 'BlackDesertM_JP', 'colopl_quiz', 'lifeafter_game', 'Line2Revo', 'Ura0817V', 'Kaerumask',..					
Forum, peing, white cat, delivery, question, Miratibu, guild, the day before ratio, wilderness, ww, char..					
質問箱, peing, 白猫, 配信, 質問, ミラティブ, ギルド, 前日比, 荒野, ww, キャラ, 荒野行動, 募集中, お願い,..					
15.00	-	238,967	1,214,983	1.677	127
'williejia', 'THECUMCONTROL', 'LanNick8', 'chenhui_hifunbb', 'Dubeyyjun', 'THEMUSCLEMANS', 'avgvchina', ..					
-..					
⊠⊠, ⊠逼, 无套, 私信, ⊠巴, 微信, 自己, ⊠源, ⊠个, ⊠吧, 哈哈, ⊠⊠, ⊠哥, 菊花, 爸爸, ⊠候, 关注, 老..					
15.01	-	97,650	367,369	2.342	220
'gayfetishjp', 'trance_video_', 'BEASTMUSCLEMAN', 'THEMUSCLEMENS', 'HUNK_CHANNEL', 'KENTA..					
-..					
私信, ⊠⊠, 自己, 大家, 哈哈, 需要, 筋肉, 歡迎, 有人, ゲイ, 沒有, 勃起, ノンケ, 微信, 朋友, 什麼, 高画質,..					
15.02	LGBT - Friends	77,760	1,032,481	4.589	1,718
'tarseitwain', 'dsuke1120', 'pleasure_yuta', 'kaniumeeee', 'ajinoli_s31', 'You_yan', 'Taaaiiiyooo', 'Y_c..					
Open, wait, Forum, peing, coming to the store, sales, EmiEmi, open, 20 o'clock, work, love, Mom, ww, que..					
オープン, お待ち, 質問箱, peing, 来店, 営業, 笑笑, open, 20 時, 仕事, 好き, ママ, ww, 質問, 21 時..					
15.03	LGBT - Adult contents	76,117	339,304	3.162	428
'danshizukan', '_BOYSTUDIO_', 'xiroqui', 'nyunnnnn7', 'muscle_justice_', 'treetree_884', 'GT_LINE_X', 'k..					
Gay, preparedness, block, dm, erotic, Straight, videos, penis, Forum, men's, peing, erotic, EmiEmi, Mast..					
ゲイ, 覚悟, ブロック, dm, エロ, ノンケ, 動画, ちんこ, 質問箱, 男子, peing, エロい, 笑笑, オナニー, イケ..					
15.04	LGBT - Anime	67,005	889,991	4.063	592
'hyaku1063', 'pcste5fje', 'okmt_RedBull', '4jhapp_lw', '41raco', 'sdorica_rayark', '12beat13', 'inusimah..					
Samo, nsfw, character, furry, love, jmof, Beastman, fursuitfriday, compared with the day before, 18, new..					
サモ, nsfw, キャラ, ケモノ, 好き, jmof, 獣人, fursuitfriday, 前日比, 18, 新刊, 感じ, ケット..					
15.05	-	58,246	237,051	2.132	173
'toumodjb', 'xudazhuangdeha1', 'petercao518', 'wxq924482497', 'heicuda', 'mengguxiong1', 'taiyo3313', 'X..					
-..					
⊠巴, ⊠⊠, 微信, ⊠源, 需要, 自己, ⊠个, 爸爸, 哈哈, rush, ⊠⊠, 私信, 无套, ⊠是, 老婆, 你⊠, 朋友,..					
15.99	LGBT - Others	18,871	36,410	3.060	661
'latinboyzxxx', 'POLOPLUTO1', 'Khai_kni', 'orgyologoII', '2nd_streettt', 'ta2ya_1030', 'DickCuck', 'asix..					
Kun, gay, utau, date, sm, inquiry, nicovideo, shuffle, school, going to work, boy, bot, boy, master, men..					
ｸﾝ, ゲイ, utau, date, sm, 問い合わせ, nicovideo, シャッフル, 学園, 出勤, ボーイ, bot, bo..					
16.00	-	104,464	338,635	2.148	1,475
'VOAChinese', 'RFA_Chinese', 'bbcchinese', 'nytchinese', 'PDChinese', 'dw_chinese', 'fangshimin', 'remon..					
-..					
中国, 美国, 香港, 自己, 什么, 你⊠, 中共, 国家, ⊠个, 民主, 哈哈, 政府, 人民, 如果, ⊠在, ⊠⊠, 中國, 自..					
16.01	-	91,599	1,167,883	1.662	2,090
'TuCaoFakeNews', 'IntyPython', 'LifetimeUSCN', 'Xybaiyun2018', 'ttingxiao', 'japanaqi', 'cindywei2017', ..					
-..					
香港, 中共, 美国, 中国, 人民, 自己, 国家, ⊠个, 什么, 政府, ⊠⊠, ⊠党, 如果, 民主, 中国人, 你⊠, 香港人,..					
16.02	-	77,482	364,598	1.764	1,249
'lihkg_forum', 'Tonyworld15', 'MaggieHo20', 'badiucao', 'Lionmountains1', 'hklemontea', '9Ovvv', 'chenqi..					
-..					
香港, 香港人, 警察, 中國, 自己, 示威, 政府, 参加者募集, 中共, 大家, 手足, 美國, 市民, 中国, lv, id, 参..					
16.03	-	70,758	379,808	2.596	1,355
'cni9k', 'nishuang', 'Wcwya', 'williamwoo7', 'williamlong', 'sanzhao4', '0792z', 'KenWong_', 'hu_lalalal..					
-..					

自己, 什么, 哈哈, ☒个, 中国, ☒在, ☒是, ☒候, 美国, 香港, 如果, ☒☒, 怎么, ☒得, 所以, 公司, 不能, ☒..					
16.99	-	239,462	695,098	3.538	622
'bindarsou', 'feituji1994', 'gowKE4HReZ7ZBc5', 'TW_nextmedia', 'OttoHuang120', 'RuthMiller916', 'initium..					
-..					
香港, 自己, 台灣, 暴徒, 中國, 什麼, 沒有, 中国, ☒子, 美国, ☒的, ☒☒, ☒党, 不要, 支持, 政府, 如果, 所..					
17...	"NGT46"	413,515	5,202,410	2.578	347
'nogizaka46', 'keyakizaka46', 'nogisatsu', 'otome_kagura', 'nogikoiofficial', 'mechakari', 'hinatazaka46..					
-..					
乃木坂 46, 乃木恋, 欅坂 46, 軍団, 日向坂 46, 乃木坂, 乃木, 西野七瀬, 齋藤飛鳥, id, 自発, 飛鳥, 写真集, 乃木..					
18...	"Identity "	411,287	4,711,302	2.331	810
'IdentityVJP', 'IdentityV_info', '5tukirin', 'Rcollection_PR', 'MotimotiPopo', 'nyulouis', 'wolf5rin', '..					
-..					
人格, identityv, ハンター, 応募, シノアリス, ラン, イラスト, ジョゼフ, チェイス, リッパー, 質問箱, 好き, ..					
19...	figure skating	266,702	3,316,463	1.928	1,420
'YoshikiOfficial', 'HydeOfficial_', 'asahi_photo', 'KinbakuTw', 'mainichiphoto', 'SUGIZOofficial', 'sn_f..					
-..					
羽生, 選手, 羽生結弦, フィギュアスケート, 昌磨, yoshiki, hyde, 結弦, 高橋大輔, 宇野昌磨, 大輔, 演技, ス..					
20...	"Dragon Ball Legends"	163,273	1,276,171	3.418	1,183
'Nenaro_YouTube', 'iiwaneOBASANwww', 'Siitake_Oisii', 'noa_dbh', 'mnlselvvigets5', 'amour_macherie', 'he..					
-..					
なりきり, bot, r1, ふふ, 反応, 貴女, お迎え, 大丈夫, 仕様書, 好き, すり, きっと, ドッカンバトル, 苦笑, 前..					
21...	"Pokemon(Go)"	134,001	1,410,747	3.983	916
'PokemonGOAppJP', 'gamewith_pkgo', 'pokemongo_db', 'DFF_OperaOmnia', 'ekimemo', 'YAMADA_N..					
-..					
ポケモン go, 色違い, ロマサガ, レイド, 駅メモ, 三国志大戦, ポケモン, ポケ, rs, キャラ, イベント, go, フレンド..					
22...	Online Radio	68,672	549,142	2.697	3,367
'spoon_radio', 'sushizanmaikabu', 'Tomato556931', 'Shika_Barten', 'bacon_kirai', '294_virtual', 'cana1a7..					
-..					
質問箱, peing, 配信, spoon, 質問, reality, 笑笑, 匿名, 好き, 募集中, ww, lt, 大丈夫, スプー..					
23...	Community of teanage girls	54,371	240,738	2.349	223
'Rabi________oO', 'mm_skm9', 'MiuPima', '6ai5ueO', 'O27NO', 'nynkfktm', 'DtyVi', 'l93Ott', 'typhoon_____y', '..					
-..					
情報, 傘下, ばら, お願い, 核酸, 企画, 規格, 無償, 協力, 酸化, 初心者, 出戻り, 当方, dm, 失礼, ho, スパ..					
24...	-	52,183	98,067	2.447	252
'SU44O', 'alghada_k33', 'printpuncakalam', 'en_haakt', 'waleedalfarraj', 'AhmedHNBO', 'FAIZ___311', 't9j_..					
-..					
ff, special, repost, takip, destek, modoactivo, de, bir, this, the, ˚..					
25...	Male musical group "9bic"	50,758	331,904	2.721	511
'konyafes2017', '9bic_official', '9bic_satsuki', 'hare_suta', 'ONEDAY_RYOMA', '9bic_minato', '9bic_ryoga..					
-..					
現場, チェキ, 甘党, live, 楽しみ, 物販, すき, 好き, だいすき, 質問箱, jol, 生誕, 幸せ, ワンマン, 公演,..					

Supplementary Table S3: The information spread from the top 100 influencers and the top 100 co-reply/retweet users

This table shows the information spreading (the number of replied or retweeted) destinations from the top 100 influencers and the top 100 co-reply/retweet users. The destination users are grouped as: left EC, right EC, and others. We found that the information destination from both the top influencers and co-reply/retweet users are highly polarized. The latter is polarized compared to the former. Both ECs have a similar tendency. This result indicates that co-reply/retweet authors do not influence the community, but greatly influence the EC.

Number of replies/retweets from the left EC, right EC, and other communities of the left/right ECs' influencer and co-reply/retweet cores. The values given in parentheses are the ratio of each group' s information spread target.

	Left EC	Others	Right EC
Left influencer	83,695,320 (0.8411)	13,625,150 (0.1369)	2,186,360 (0.0220)
Left co-reply/retweet core	14,017,010 (0.8814)	1,703,550 (0.1071)	183,420 (0.0115)
Right influencer	1,851,910 (0.0148)	28,518,810 (0.2282)	94,595,570 (0.7570)
Right co-reply/retweet core	108,420 (0.0077)	1,644,200 (0.1174)	12,258,130 (0.8749)

Supplementary Figure S1: The relationships between the number of retweets/replies and the number of retweeted/replied to for each user in both ECs

Both ECs have highly influential cores that are retweeted/replied to many times. We examined the engagement of retweets/replies. We found a moderate correlation (0.54 in the left EC and 0.58 in the right EC) between the number of retweets/replies and the number of retweeted/replied to for each user in both ECs. With the exception of some highly influential users who have a lower retweet/reply engagement rate (such as members of parilament), most influential users tend to engage actively.

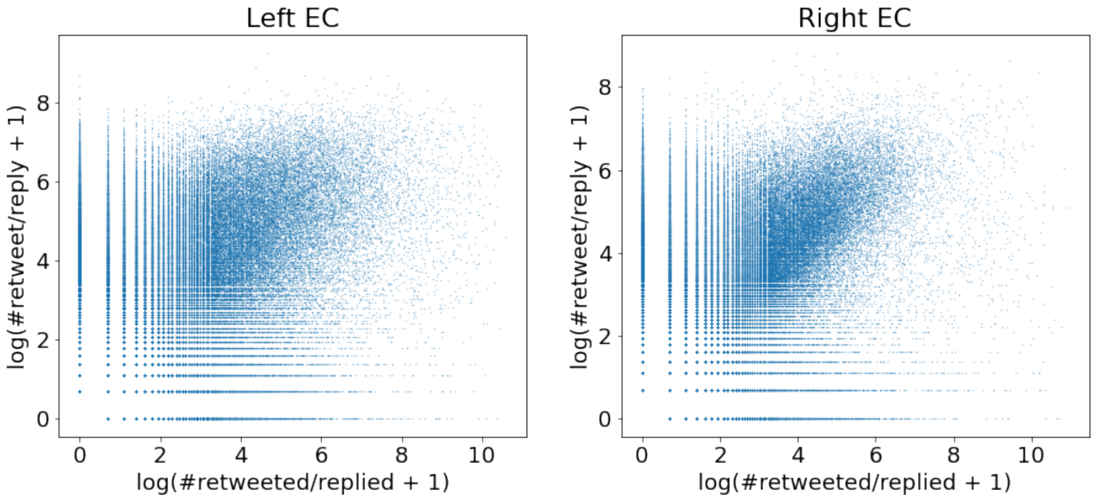

The relationships between the number of retweets/replies and the number of retweeted/replied to for each user in both ECs.
